# Sports and Child Development

**DOI:** 10.1371/journal.pone.0151729

**Published:** 2016-05-04

**Authors:** Christina Felfe, Michael Lechner, Andreas Steinmayr

**Affiliations:** 1 Swiss Institute for Empirical Economic Research, University of St. Gallen, St. Gallen, Switzerland; 2 Center for Economic Studies and ifo Institut, Munich, Germany; 3 Center for Economic Policy Research, London, England; 4 Policy Studies Institute, London, England; 5 Institute for the Study of Labor, Bonn, Germany; 6 Institut for Employment Research, Nuremberg, Germany; 7 Department of Economics, University of Munich (LMU), Munich, Germany; TNO, NETHERLANDS

## Abstract

The role of curricular activities for the formation of education, health and behavioural outcomes has been widely studied. Yet, the role of extra-curricular activities has received little attention. This study analyzes the effect of participation in sports clubs—one of the most popular extra-curricular activities among children. We use alternative datasets and flexible semi-parametric estimation methods with a specific way to use the panel dimension of the data to address selection into sports. We find positive and robust effects on children’s school performance and peer relations. Crowding out of passive leisure activities can partially explain the effects.

## 1 Introduction

The importance of people’s cognitive skills, such as the performance in school in explaining socio-economic success is widely acknowledged (see for instance [1,2]). Recently, non-cognitive skills, such as behaviour or emotional strength, have received increased attention when analysing the determinants of socio-economic success (see for instance [3]). Health, moreover, is a prerequisite for any educational and professional achievements (see for instance [4,5,6,7,8]). As established by the developmental literature in economics as well as the epidemiological literature, the fundaments for health and socio-economic success are laid out early in life (see, for instance [8,9,10]). Yet, while a substantial body of literature examines the role of the education and health system for the formation of children’s health, education and behaviour (for an overview please refer to [11]), the relevance of extra-curricular activities for children's development, is not well understood.

One of the most popular extra-curricular activities among children is sports. According to the National Alliance for Youth Sports (NAYS), approximately 65% of children worldwide are involved in sports activities. While 55% of American children are involved in youth sports, among German children who are the target of this analysis, this number is somewhat higher: about 70% of all children aged 6–14 engage in sports activities [12]. Moreover, many countries support such activities with sizeable public subsidies.

Despite the popularity of sports as a leisure activity, there exists only little empirical evidence on the relation between sports participation and children's development. Prior research, mostly stemming from paediatrics or psychology, has devoted much attention to the role of sports for children’s physical and mental health. Strong et al. [13] provide a summary of the huge literature in this field. Examples of more recent studies are Morrow et al. [14] who in an observational study find that obeying the levels of physical activity as recommended by the national and international guidelines provides clear health benefits. While this study concentrated on fitness outcomes, another example of this large literature is an experimental study of a school-based physical activity intervention for which Meyer et al. [15] find positive effects for bone development during childhood. Research on the link between sports and education has mainly focused on the role of sports activities among high school students or adolescents. An exception is a recent study by Dills, Morgan & Rottbof [16], which studies the impact of recess and physical education on primary school children’s learning process. A positive link between participation in high school sports and educational attainment, peer relations and professional success is well established [17,18,19,20,21]. Yet, the underlying mechanism is not well understood. Rees & Sabia [22], for instance, hardly detect any improvement in university students' overall grades and only a modest impact on students' educational ambitions. Thus, the question when and through which mechanisms sports exerts its influence on people's educational and professional success remains open.

When addressing this question it is crucial to bear in mind that health, education, behavioural and emotional skills acquired early in life reinforce their development later in life and thus may partially explain later socio-economic success [23]. For this reason, it is interesting to understand whether sports participation early in life might help shaping children's health, education and behaviour. We therefore analyze the impact of sports participation during preschool and primary school on outcomes related to these dimensions.

To be more precise, we focus on participation in sports clubs among children aged 3 to 10 years in Germany. In Germany, sports clubs are the key institutions organizing sports activities for children. In contrast for example to the U.S., where youth sports is heavily organized in schools, in Germany most child and youth sports, both for leisure and competition, is organized in clubs (according to the German Olympic Association (Deutscher Olympischer Sportbund, 2009), 76% boys and 59% girls aged 7 to 14 are doing sports in a club). Schools play only a minor role in offering extra-curricular sports activities: in Germany, physical education in primary school is regulated by law and forms part of the mandatory curriculum, but supply of further sports activities offered by the school is rare. Focusing on sports participation in clubs instead of physical activity in general, which might also include unguided sports or play, has the advantage that its content as well as its objectives can be more clearly defined. Obviously, sports club participation may still contain large variation in the type of activities children engage in and in the environment they face. As such, sports club participation acts as a “social address” as described in Bronfenbrenner [24]. Addressing this variation, however, is beyond the scope of this paper. In addition, self-reported participation in sports clubs may be less prone to reporting bias than self-reported physical activity in general. Reporting bias for self-reported physical activity in general may occur in particular, if parents answering these questions would like to be considered as being 'responsible and caring' and thus report socially desirable levels of physical activity. It is also important to note that the reported level of physical activity *outside* of sports clubs is comparable across children participating in sports clubs and children *not* participating in sports clubs (see Section 5.2 for details)

Our study relies on two alternative datasets. First, we use the "German Health Interview and Examination Survey for Children and Adolescents" (henceforth KiGGS), a cross-sectional (medical) survey for Germany, which contains information on a wide array of children's objective health, education and behavioural measures (5,632 children). This rather large sample allows for a heterogeneity analysis. Second, we take advantage of the German Child Panel (henceforth GCP) which provides us with comparable information for 1,449 children at two points in time and thus allows for addressing the issue of endogenous selection into sports in more detail.

The major challenge for any empirical study focusing on this topic is the inherent selection problem. Problematic selection issues may arise if parents, who are more concerned with the development of their children, are more likely to send their children to sports activities. Of course, such parents are very likely to exhibit further characteristics that foment their children’s development per se. One further concern may be the potential correlation between sports participation, enhanced through a well-developed sports infrastructure, and exposure to a further development-stimulating environment, e.g. better school quality, a more sophisticated health system or more recreational areas. In our study, we argue that the very detailed information on background characteristics as well as a set of regional fixed effects makes a selection-on-observables strategy credible. This strategy is based on the conditional-independence-assumption, which states that conditional on child, family, and regional characteristics, observed differences in child outcomes between children participating and not participating in sports clubs, is due to participation. We employ a semi-parametric matching estimator that matches treated (participating) and control observations (not participating) based on a wide range of observable characteristics. We scrutinize the claim of conditional independence by testing whether our estimates are sensitive to including simulated unobserved confounding variables. To further challenge the underlying assumption of our baseline strategy, we correct for selection into sports and reversed causality by exploiting the longitudinal nature of the GCP and controlling for lagged outcome variables as well as past sports status.

Results based on the selection-on-observables strategy point towards a positive impact of sports on children’s health, school performance and behaviour, which are consistent across both datasets. Yet, once we consider lagged outcomes and past sports club participation, we detect only significant effects on children’s school performance and on one dimension of children’s behaviour: overall school grades improve by 0.19 standard deviations (henceforth sd), while peer problems decrease by 0.22 sd. We also provide evidence that a reduction in TV consumption among children participating in sports clubs may in part explain our results.

Our paper is most closely related to two recent papers by Cawley, Frisvold & Meyerhofer [25] and Dills, Morgan, & Rotthoff [16]. Both studies focus on physical education as part of the curriculum in primary school. The focus of the first paper lies on the impact of physical education on the prevalence of obesity, while the second paper concentrates on the impact of recess time and physical education on children’s learning outcomes. On the contrary, our focus lies on the impact of sports club participation among children aged 3–10 years on children’s health, school performance and behavioural development. As such, our paper does not study the impact of physical activity per se, but the impact of, in the terminology of Bronfenbrenner’s [24], a “social address”. In other words, sports club participation is the object of study, but what happens inside a sports club is largely unknown. Section 5.3 provides a discussion of the likely activities children may be exposed to when participating in a sports club and thus on the mechanisms through which sports club participation may exert its effects on children’s health, education and behaviour.

The reminder of the paper is structured as follows. The next section describes briefly the organization and the financing of sports-related activities in Germany. Section 3 introduces the data and provides descriptive statistics for the samples used in this study. Section 4 explains our identification and estimation strategies. Section 5 presents the results and discusses effect heterogeneity as well as likely mechanisms at work. Section 6 concludes and discusses the policy relevance of our findings. The Supporting Information to this paper as well as an internet appendix (please refer to www.sew.unisg.ch/lechner/kispo) contain additional information on the data and the estimation.

## 2 Institutional Background

Doing sports is the second most popular leisure activity among German boys: 59% of all boys indicate that spending time with their best friend is their favorite leisure activity, closely followed by doing sports (53%). For girls, doing sports ranks still among the most popular leisure activities, behind spending time with friends or listening to music, but only 33% of the girls consider doing sports as their most preferred leisure activity [26].

Regular participation in sports club among children is rather high (see Table 1). The engagement in sports clubs rises steadily until age 8/9 (from 25% for the 3-year-old boys and 29% for the 3-year-old girls, to 71% for the 9-year-old boys and 62% for the 8-year-old girls). On average participation rates in sports clubs among preschool and primary school age children amount to 56% among boys and 54% among girls. Comparing these rates with overall physical activity rates (77% among boys and 75% among girls) reveals that sports clubs constitute the major institution where children practice sports.

**Table 1:** Participation in sports clubs. Note: The numbers presented above are based on our own calculations using the KiGGS data. They represent the share of children in each age group reporting attending a sports club at least once per week.

Age	Participation	
	Explicitly in Sports Clubs	
	Male	Female
3	0.25	0.29
4	0.32	0.42
5	0.44	0.50
6	0.54	0.51
7	0.62	0.56
8	0.67	0.62
9	0.71	0.58
10	0.66	0.55

The participation rates based on our dataset resemble closely official registrations in sports clubs. The German Olympic Association (Deutscher Olympischer Sportbund, 2009) for instance, reports club participation rates of 76% among 7–14 year old boys, and of 59% among 7–14 year old girls. Clubs serve as site for the most popular sports. Boys' favorite sport, soccer, is exercised by 45% of all boys aged 7–14, followed by gymnastics (14%), tennis (5%), handball (5%), and athletics (5%). Girls' favorite sports are gymnastics (37%), soccer (11%), horse riding (8%), athletics (7%), and swimming (6%). Thus, sports club participation may capture an important part of children’s level of physical activity.

The high rates in sports club participation may be in part due to rather low membership fees, which vary between 0 and 120 Euro per year for children, and 0 and 150 Euros per year for adults. Reductions in the membership fee for whole families participating in a sport club are common. Moreover, social assistance frequently bears the membership fees. In other words, exclusion based on financial grounds hardly seems to be an issue.

## 3 Data

The empirical analysis draws upon two different datasets. The first dataset is the "German Health Interview and Examination Survey for Children and Adolescents" (henceforth KiGGS), which is a comprehensive, Germany-wide, representative interview and examination survey for the age group 0–17 years. Between May 2003 and May 2006 17,641 participants were interviewed and examined [27]. The second dataset is the "German Child Panel" (henceforth GCP), which includes observations of 2,709 children up to three times. The first interview took place in 2002, when children were between 5 and 8 years old, the third and last interview took place in 2005, when children were consequently between 8 and 11 years old.

The KiGGS dataset includes objective measures of children's health as well as subjective measures regarding children's school performance and behaviour. Crucial for our analysis is also the information on children's sports activities. Additionally, KiGGS provides us with rich information on the family background, such as demographic features, socio-economic characteristics, and measures for parenting styles. Finally, based on the individual location of residence, we can add a set of regional characteristics available in the INKAR database (Indikatoren und Karten zur Raum- und Stadtentwicklung, see http://www.bbsr.bund.de for more details). Thus, we can study the relation between children's sports participation and their health, education and behavioural development while conditioning on a rich set of potentially confounding variables, i.e. factors, which simultaneously influence children's participation in a sports club and children's development.

Due to its longitudinal nature, the GCP enables us to tackle the issue of selection into sports due to time constant personality and environmental features. We use sports participation and outcome measures from the second wave and take advantage of the first wave exclusively as a source for control variables, such as lagged outcome measures, lagged sports participation, as well as individual and family background characteristics (see Section 4 for details).

Our initial sample from the KiGGS data consists of 8,023 children in the relevant age range (in either preschool or primary school). Due to missing information on the individual participation in a sports club (325 observations), our sample is further restricted. Additionally, we exclude all foreigners from our analysis (1,025 observations), because some ethnic groups can be expected to behave differently in terms of engagement in social activities and in particular in sports activities (especially when their child is a girl). Notice that the classification of “foreigner” depends on the country of birth and the origin of the parents, an exact definition is available in the documentation of the KiGGS database (http://kiggs.de/service/English/index.html). Requiring the availability of the information about children's health, education and behavioural development as well as on the residential neighborhood, our final sample contains 5,632 children.

Employing the same approach when defining our sample based on the GCP, 1,449 children remain. Due to its much larger sample size as well as the superior quality of the health measures, the KiGGS data serves as our main dataset. Thus, if not mentioned otherwise the following empirical analysis refers to the KiGGS data. Yet, we create outcome measures, treatment, and control variables in the GCP analogously to the respective variables in the KiGGS data (with minor exceptions).

Concerning the information on sports, parents answered a question about the frequency with which their child was performing sports activities in a club. They could choose between 5 different categories: "never", "less than once per week", "once or twice a week", "3–5 times and a week" and "almost daily". Table 2 shows that there are two groups of children: those who do not join a sports club on a regular basis (45%) and those who attend at least once a week a lesson in a sports club (55%). Consequently, we aggregate this information and distinguish between participating regularly in a sports club (at least once per week) and not participating regularly in a sports club (less than once per week).

**Table 2:** Frequency of participation in a sports club. Note: Computed from our estimation sample of KiGGS.

	Sports in a Club	
Frequency	Observations	Share in %
More than 5 times/week	50	1
3–5 times/ week	331	6
1–2 times/ week	2,723	48
Less than once per week	330	6
Never	2,198	39

The KiGGS data contains a large amount of health-related information. Based on a physical examination, it provides objective measures for children's height, weight, skinfold (examined at the back), and the resting pulse rate. Additionally, in a questionnaire parents are asked to rank the health status of their child choosing on an integer scale from 1 to 5, where 1 indicates very good health and 5 a very bad health.

Parents report their children’s school grades, which ranges from 1 ("very good ") to 5 ("bad "). We average across the grades reported for Math and German and create the unweighted average. Notice that information about academic performance is only available for children in primary school. Moreover, the age when children start receiving grades varies across federal states. Thus, the number of observations with information on children’s school performance is considerably lower than the total sample size (1,698 children).

The questionnaire also includes 25 questions to allow for a screening of children's behavioural outcomes. These questions belong to the Strength- and Difficulties Questionnaire (SDQ), a behavioural screening device developed by Goodman [28]. The SDQ, which can be found together with the scoring information in the S4 and S5 Appendices, has been validated and rated as a very reliable tool to gauge children's emotional symptoms, conduct problems, hyperactivity, peer relationship problems, and pro-social behaviour [29,30]. Each score ranges from 0 to 10, with 0 indicating no problems and 10 indicating severe problems in the respective dimension. The total difficulties score corresponds to the sum of the first four dimensions.

For interpretational convenience and comparability with the findings of existing studies, we compute z-scores by standardizing all outcome measures to mean zero and variance one. Such standardization is not only common in the medical literature but also allows for comparison with the findings of the previous literature. S5 and S6 Tables provide descriptive statistics as well as estimations results using measures in levels.

Finally, to allow for a homogenous interpretation across all dimensions we also invert the pro-social score and call it antisocial behaviour. Thus, generally for all indicators presented lower values signify a better performance of the child. Table 3 displays descriptive statistics for all outcome measures.

**Table 3:** Descriptive statistics for children's health and human capital. Note: All outcome variables are standardized to mean zero and variance one. A lower value corresponds to a better outcome. The lower number of observations for grades appears because not all children are enrolled in school and not all school-age children receive grades. P-values stem from two-sided t-tests comparing the means for children doing and not doing sports in a club. Descriptive statistics of the unstandardized variables can be found in S6 Table.

	No Participation in Sports Clubs	Participation in Sports Clubs	Sports—No Sports		Obs.
	Mean	Mean	Difference	p-val. %	
**Health**					
Subjective Health	0.07	-0.06	-0.13***	*0*	5,632
BMI	0.00	0.00	0.01	*75*	5,632
Skinfold	0.05	-0.04	-0.08***	*0*	5,632
Pulse	0.20	-0.16	-0.36***	*0*	5,632
**School Performance**					
Overall Grade	0.18	-0.10	-0.28***	*0*	1,698
**Behavioural outcomes**					
Emotional Problems	0.06	-0.05	-0.10***	*0*	5,632
Behavioural Problems	0.08	-0.06	-0.14***	*0*	5,632
Hyperactivity	0.10	-0.08	-0.18***	*0*	5,632
Peer Problems	0.14	-0.12	-0.26***	*0*	5,632
Overall Score	0.13	-0.11	-0.24***	*0*	5,632
Antisocial Behaviour	0.07	-0.06	-0.12***	*0*	5,632

*** denotes statistical significance at the 1% level.

Table 3 shows that the means of the BMI of children participating in a sports club and children not participating in a sports club are comparable. Yet, the BMI constitutes a rather poor measure for children's tendency to be overweight [31]. Skinfold, a more reliable measure for children’s body fat, is significantly lower among children participating in a sports club (-0.08 sd). Children joining a sports club have on average also a significantly lower resting pulse rate (-0.36 sd). Moreover, their parents rate their health significantly better (-0.13 sd). Children performing sports in a club also perform generally better in school. The difference between children engaging in sports versus children not engaging in sports amounts to -0.28 sd. Finally, physically active children score also significantly lower in the strength and difficulties questionnaire (-0.24 sd), implying that they are less hyperactive and have fewer peer, emotional, behavioural or conduct problems than physically inactive children. Moreover, children enrolled in a sports club report less antisocial behaviour.

Taken together, the raw differences draw a clear picture: children participating in a sports club outperform children not participating in a sports club in all dimensions. Yet, these unconditional comparisons do not address the concern that differences may reflect sorting of children with a priori better conditions into clubs (see for example Stattin and Kerr [32] for an extensive discussion on self-selection into leisure activities). In fact, the background characteristics of the two groups show substantial differences (see S1 Table). Active children are older and taller; their parents are better educated, more likely to be working and to engage with their children; their families are more likely to belong to a better social class and to live in urban areas. These differences highlight the importance of conditioning on potentially confounding variables when analyzing the impact of sports participation on children's development.

This study is based on de-identified survey data collected by the "Robert Koch Institute" (KiGGS data) and the "German Youth Institute" (GCP data). The KiGGS Study was approved by the Charité/Universitätsmedizin Berlin ethics committee and the Federal Office for the Protection of Data and was conducted according to the Declaration of Helsinki (http://www.kiggs-studie.de/deutsch/studie/kiggs-im-ueberblick/datenschutz-und-ethik.html). Data collection by the German Youth Institute was conducted following the ethic codex of the German Society for Sociology, the German Society of Psychology, and the German Society for Education Research, among others (http://www.dji.de/index.php?id=43427&L=0). The institute’s scientific advisory board reviewed the GCP for compliance with scientific and ethical standards. As the authors worked only with the anonymized data and were not involved in data collection, no additional IRB approval was required for this study.

## 4 Conceptual Framework and Econometrics

This section clarifies what we mean by the causal effect of sports club participation on children’s development, discusses the assumptions underlying our identification strategies, and introduces the different empirical strategies.

We define the causal effect of sports club participation on a child's health, education and behaviour (outcome) as the difference of the outcome in case the child participates in a sports club on a regular basis and the outcome the same child would have, if she would not participate in a sports club on a regular basis. Obviously, sports club participation may encompass a variety of activities and the effect of sports club participation may work through different mechanisms. While distinguishing between the underlying mechanisms is beyond the scope of this paper, we devote some time discussing activities that come along with exercising sports in a club as well as counterfactual activities—the activities children spend less time on when participating in a sports club—in Section 5.3.

### 4.1 Identification

The previous section highlighted the need to take selection into sports seriously. We therefore employ first a selection-on-observables strategy using the KiGGS data that eliminates differences in observable confounding factors. Second, we corroborate our results using the simulation-based approach by Ichino, Mealli and Nannicini [33], which assesses the sensitivity of our baseline matching estimates with respect to potentially missing confounders. Third, we exploit the panel structure of the GCP. Before explaining the respective estimation methods, we discuss the identifying assumptions underlying the different empirical strategies.

The main identifying assumption of the selection-on-observable strategy is the so-called *Conditional Independence Assumption* or *No Confounding Assumption*—henceforth CIA [34]. The CIA requires that potential outcomes (the outcome that would be realized if a child were exposed to a particular treatment) and treatment are independent conditional on a set of suitable observable characteristics. In other words, we need to condition on all variables, which simultaneously determine children's development and children's participation in a sports club. As will be explained later, we do so by matching treated children with untreated children who have the same observable characteristics. Theory and empirical evidence on the determinants of children's development are the basis for the selection of these variables.

According to the seminal work by Leibowitz [35] the investments made by parents, school, and social environment determine children's health, education and behavioural development. Empirical research has put forward the following determinants of children’s development: families' socio-economic status [8,11,36,37], parental education [38], neighborhood [39] and children's initial endowments [38]. The psychological literature has also put forward the relevance of parents' attitudes and parenting practices for their children's development [40].

Given that a similar set of factors is likely to influence children’s probability of participating in a sports club, we control for a comprehensive set of child, family and regional characteristics. The following blocks of variables mean to proxy these three dimensions. With respect to children’s characteristics, we consider the following information. We use birth weight as a proxy for a child's health status early in life [11]. Besides age and gender, we also condition on children's height, which is associated with higher levels of sports participation as well as better outcomes later in life [41]. To describe the family background, we include several measures for a family's socio-economic status, such as parental education, labour force participation and occupation, household income and an aggregated index for socio-economic status. While we lack information about parents' own physical activities, we use parents' BMI to approximate their physical fitness. Furthermore we include a broad range of measures for parenting style, such as the enforcement of rules or how much family members care about each other. We supplement the latter variable block by information about how often the child brushes its teeth and whether the mother smoked during pregnancy.

Further factors determining children’s health, education and behaviour are the quality of the school and health system as well as further amenities. In case children’s sports participation correlates with exposure to a better education or health system or further development enhancing amenities, our estimated effects of sports participation may be upward biased. In Germany, education as well as culture and sports are under the responsibilities of the states, the so-called Länder (cf. Art. 30 of the German constitution). As a consequence, we observe differences in the school infrastructure and curricula as well as in the public funding for sports mainly at the state level. In order to tackle any potential bias arising due to endogeneity of sports and further infrastructure promoting children’s development, our main analysis controls for state fixed effects. In addition, we use several measures of regional characteristics such as municipality size, availability of recreation areas, tax income of the municipality, employment structure and population development. Controlling for these additional regional features allows us to address differences at the municipality level that go beyond differences in state regulations.

Given the richness of our dataset, which does not only provide us with the usual information on children’s individual and family background characteristics, but also includes detailed information on the home environment and parenting practices, we believe that the CIA is credible in our context. Nevertheless, additional estimation strategies help us to provide further evidence for the robustness of our results.

In particular, we address potential concerns related to the cross-sectional character of the KiGGS data. One concern and source of a potential bias is the problem of reverse causality. Children a priori endowed with better health, school performance or behaviour capital might be more likely to engage in sports. Employing longitudinal data, such as the GCP, and conditioning on lagged values of all outcome variables, can remove much of the resulting bias (cf. Lechner and Wunsch [42]). Moreover, existing theories on skill formation postulate that children’s development is a cumulative process [10]: early inputs foment children’s health, education and behavioural development, which in turn boost children’s later development. Thus, lagged outcomes serve as a proxy for prior inputs, both observed and unobserved ones. As a result, comparing GCP results with and without lagged outcome variables enables us to assess the sensitivity of the estimates to their exclusion. Notice, that this strategy also substitutes the previously current control variables by lagged values of the controls variables.

Yet, controlling for lagged outcome and control variables may not be enough. If there are persistent components in sport activities, as is likely, past control variables, such as lagged outcome variables or parenting style, may already be influenced by past sports activities and thus ‘mask’ some of the effects of sports participation. Consequently, our results for the effects of sports participation may still be biased even if controlling for lagged control variables. To tackle this issue we implement the strategy suggested by Lechner [43]. This strategy proposes to restrict the sample to children who in period 1 do not engage in any sports activity and then to analyse the effect of their sports participation in period 2 on outcomes in period 2. Doing so removes the endogeneity problem: by construction, covariates cannot be differentially influenced by sports participation in period 1 as no child engages in sports in period 1. Notice, that this strategy again controls for the full set of lagged outcome variables as well as lagged control variables.

The strategies suggested address the issue of endogeneity due to unobservable time constant characteristics, not, however, due to potentially unobserved time varying shocks that occur after outcomes are last observed. While the only remaining threat to identification are unobserved leaps in children’s development, which simultaneously stimulate or dampen their participation in a sports club, we cannot fully exclude their existence. We therefore assess the sensitivity of our baseline matching estimates with respect to potentially missing confounders using the simulation based approach suggested in Ichino, Mealli, and Nannicini [33] and Nannicini [44]. The underlying idea is to test the sensitivity of the results to the inclusion of a simulated confounding variable which is related to the potential outcomes and the treatment. In contrast to alternative approaches (e.g. [34,45,46,47]) this method tests the sensitivity due to unobserved confounders in the presence of already existing control variables and does not introduce any new restrictions, for example in the form of a requirement to model the relationship between outcomes, treatment, and confounders. For details on the method and its implementation, please refer to S3 Appendix.

In further analyses, we also exploit individual distance to the closest sports facility as an instrument for children’s sports participation. Unfortunately, the first stage—the impact of distance to the closest sports facility—works only in certain geographical regions—namely on the countryside (please refer to Steinmayr, Felfe & Lechner [48] for more details on the first stage). As a result, estimates based on an instrumental variable method are too imprecise to serve as a useful robustness check. Yet, the interested reader can find these estimation results in an earlier version of this paper (cf. [49]).

### 4.2 Estimation

Since we argued above that controlling for a vast array of potentially relevant confounding factors identifies the average effect of sports club participation, a matching estimator is a natural choice to avoid unnecessary biases coming from potentially incorrectly specified parametric models. Two central concerns with parametric models such as multivariate regression are a) the possibly incorrect linearity and effect homogeneity assumptions and b) the possible extrapolation to regions without common support. The latter assumption is particular worrisome as it implies that observations with no reasonably similar characteristics are used for comparison. Any matching estimator relies on the comparison of children who participate and who do not participate in a sports club and who are similar in their observable characteristics. A way to guarantee "similarity" in observable characteristics is to condition on an estimate of the conditional participation probability, also called propensity score [34]. Here, we follow the convention in the literature and use a binary probit model to estimate the propensity score. The full specification and the coefficient estimates for the propensity score model of our main specification are provided in S1 Table, and the results from the probit model used to estimate the propensity score using the GCP are presented in S2 Table.

Discussing the results of the propensity score model also helps providing a better understanding of how selection into participation in a sports club works. Parental socio-economic background is strongly positively associated with sports club participation. Furthermore, other measures indicate that children participating in a sports club come from more favourable family backgrounds. For example, they are less likely to have a mother that smoked during pregnancy, less likely to live in a home with mold, but more likely to brush their teeth regularly. We perform tests against misspecification (non-normality, heteroscedasticity, omitted variables), which are available upon request. The exact matching procedure used in this paper was suggested by Lechner, Miquel, & Wunsch [50] and is the one that appeared as one of the best, if not the best, matching procedure in a large scale simulation exercise by Huber, Lechner, & Wunsch [51]. This estimator is available for the software packages GAUSS, R and STATA (cf. [52]). Its matching procedure is explained in detail in the S1 Appendix.

Two issues affecting the appropriateness of matching estimators are common support and match quality. In the case of insufficient common support, we would deal with a subset of observations without appropriate matches. For this reason, we discard any observation in one state having a higher or lower propensity score estimate than, respectively, the maximum or the minimum in the other state. Moreover, to increase precision of our estimates we remove all observations with a normalized weight larger than 6% [51]. Notice that in case discarded observations systematically differ from the original sample, this selection changes the underlying population. If the common support restriction leads to a considerable reduction in sample size, one might argue that the effects are not representative for the target population any more. Fortunately, this is not a serious issue in the present study as approximately 99% of observations in our main specification and at least 91% in all our subgroup analyses are in the common support. The match quality concerns the question about the balance of the distribution of the confounders in the different treatment states. Checking the means and medians of potential confounders for matched individuals in different treatment states suggests that the after-match balance is high for all comparisons of treatment states. More information is available in the Supporting Information, which includes after-match t-statistics and standardized difference tests (see [53]) for the variables in the probit specifications as well as *χ*^2^- statistics for joint independence of the regressors and the participation state in the respective matched sample (see S10 and S11 Tables). Notice that none of the test statistics points to covariate imbalance after matching.

When exploiting the longitudinal nature of the GCP, we structure the estimation problem analogously and employ the same estimator. Notice, however, that due the rather small sample size of the GCP and the resulting loss in precision, we abstain from including state fixed effects when using the GCP. In order to guarantee comparability of our results, we re-estimate our baseline specification using the KiGGS data but exclude the state fixed effects (see Section 5.1. for details). Results do not alter significantly with and without state fixed effect (see S9 Table).

## 5 Results

The results are organized in the following way. Section 5.1 presents our main results using the KiGGS data. We then test the robustness of our estimates to including simulated unobserved confounders and to selection into sports clubs using the GCP. Section 5.2 discusses whether the effects of sports participation differ across subgroups. Section 5.3 discusses the mechanisms at work by shedding light on the activities encompassed by participation in sports clubs and on the activities actually be crowded out.

### 5.1 Main results

Participation in sports clubs during childhood has strong effects on children's health, education and behaviour. Table 4 displays the mean potential outcomes for all outcome dimensions (column 1 if participating in a sports club and column 2 if not participating in a sports club), the average effect (column 3), and the respective significance levels (column 4). Notice once again that we define all scores such that lower values imply a better performance.

**Table 4:** Matching estimates for health, school performance and behavioural outcomes (KiGGS). Note: Effect presented is the average treatment effect (ATE). p-values are computed by bootstrapping p-values of the t-statistic with 4999 replications. Note that all variables are standardized to mean zero and variance one.

	Average Outcome if Participating	Average Outcome if Not Participating	Average Effect	p-val. %
**Health**				
Subjective Health	-0.04	0.08	-0.12***	*0*
BMI	0.00	0.02	-0.01	*70*
Skinfold	-0.01	0.04	-0.06*	*8*
Pulse	-0.03	0.09	-0.12***	*0*
**School performance**				
Overall Grade	-0.07	0.06	-0.13**	3
**Behavioural outcomes**				
Emotional Problems	-0.01	0.09	-0.10**	*1*
Behavioural Problems	0.00	0.05	-0.04	*26*
Hyperactivity	0.00	0.04	-0.04	*32*
Peer Problems	-0.08	0.14	-0.22***	*0*
Overall Score	-0.02	0.11	-0.13***	*0*
Antisocial Behaviour	-0.01	0.03	-0.04	*29*

*** denotes statistical significance at the 1% level, ** at the 5% level, and * at the 10% level.

Overall, children's health is significantly better when doing sports. Parents assess the health status of their children significantly better (-0.12 sd). There is hardly any effect on BMI. This, however, does not come as a surprise as BMI might be a bad measure for child overweight and obesity. Sport participation reduces children’s skinfold (-0.06), which is a better measure for body fat. In addition, pulse is reduced significantly (-0.12 sd). The results for the different objectively measured health variables may help to eliminate a priori doubts whether subjectivity bias may drive our results. This bias would arise if parents of children who participate in a sports club systematically report a better development of their children.

Children also benefit from participating in sports activities in terms of their overall performance in school and behaviour: the overall measures of school performance and behaviour, expressed by the strength and difficulties score, both improve by 0.13 sd. A reduction in peer problems (-0.22 sd) and emotional problems (-0.10 sd) drives the latter results. Notice that in comparison to widely studied governmental interventions, such as for instance early childcare centers or targeted educational programs, these are non-negligible effects. For instance, Head Start, one of the most intensively studied educational programs in the U.S., has been shown to lead to improvements in children’s non-cognitive skills of around 0.2 sd and in children’s cognitive skills of around 0.06 sd [11]. Similarly, using the GCP, Felfe & Lalive [54] reveal an improvement in children’s non-cognitive skills by 0.1 sd after having attended a childcare centre during early childhood.

Despite the rich set of control variables, one may still cast into doubt whether we manage to take into account all determinants of children's participation in a sports club. It may be the case that children, who a priori are in better health, do better in school, or have less emotional, behavioural or peer problems, join sports clubs more frequently. To test our results for selection into treatment, we perform the robustness checks explained above.

In a first instance, we test whether our baseline estimates are sensitive to potentially missing confounders. We do so by employing the simulation-based approach by Ichino, Mealli & Nannicini [33]. For details about this method and its implementation, please refer to S3 Appendix.

Table 5 below represents the deviations of the estimates from the baseline scenario (i.e. the situation when the simulated covariate is not confounding) when including a simulated covariate resulting from four alternative simulation scenarios (scenarios 1–3). Overall, it is fair to conclude that sensitivity is very limited, as none of the deviations from the baseline scenario are significant at the 10% level. This is despite the fact that the confounders are constructed in a way to be fairly ‘harmful’. Thus, this sensitivity test suggests that our main findings are robust with respect to confounders that are in the range of what might be expected for missing confounding variables.

**Table 5:** Sensitivity check—Difference of effects under different confounding scenarios relative to the baseline scenario (ATE). Note: Each cells shows the difference in the effects resulting from an alternative confounding scenario to the baseline scenario. Scenarios 1–3 simulate the new confounding variable mimicking the relation of three important confounders to treatment and outcome, respectively (scenario 2 mimics the relation to family income > 5000 Euro; scenario 3 mimics the relation to mom’s education degree being university, scenario 4 mimics the relation to the local employment rate). Inference is based on 99 bootstrap replications and 19 draws of simulated binary confounder; quantile method, smoothed version, linear / logistic bias adjustment, symmetric p-values used. None of the differences is significant at the 10% level.

	Scenario 1	Scenario 2	Scenario 3
**Health**			
Subjective Health	0.0072	0.0129	-0.0011
BMI	-0.0029	-0.0043	-0.0060
Skinfold	0.0020	-0.0021	-0.0068
Pulse	0.0162	0.0097	0.0012
**School performance**			
Overall Grade	0.0089	0.0136	0.0060
**Behavioural outcomes**			
Emotional Problems	0.0128	0.0156	0.0058
Behavioural Problems	0.0079	0.0122	0.0035
Hyperactivity	0.0165	0.0085	0.0092
Peer Problems	0.0203	0.0189	0.0051
Overall Score	0.0204	0.0185	0.0088
Antisocial Behaviour	0.0023	0.0027	-0.0011

The remaining robustness checks draw upon the GCP data: For this reason, we first replicate our main results from KiGGS using the GCP data. Given the rather small sample size of the GCP and the resulting loss in precision, we abstain from including state fixed effects in the following series of robustness checks. The GCP lacks any objective health measures, but only provides information on children’s subjective health. We therefore rely on this measure for our robustness checks.

The comparison between column 1 (KiGGS) and column 2 (GCP A) of Table 6, which display the matching estimates applied to the KiGGS data and the GCP data, reveals that results are remarkably robust across the different datasets. We observe an improvement in children's subjective health of -0.12 sd in the GCP data, which is similar to the results based on the KiGGS data (-0.08 sd). Based on the GCP data, we also observe an improvement in children's overall school performance of -0.15 sd, and in children's overall behavioural score of -0.10 sd. Both estimates do not differ much from the respective estimates based on the KiGGS data (-0.20 sd and -0.09 sd). Moreover, the improvement in behaviour stems again mainly from the reduction in peer problems. While the reduction in emotional problems still amounts to the same magnitude, it is no longer statistically significant.

**Table 6:** Comparison of matching estimates using KiGGS and GCP. Note: The results in the first column (KiGGS) correspond to our main set of results based on the KiGGS data but not controlling for state fixed effects. GPC A to C are based on the GCP data. In GPC A we perform a pure replication of the KiGGS results where we use only the second wave of the GCP for both outcome and control variables. GCP B presents the results when we control additionally for the set of lagged outcome variables and replace all control variables by the respective control variables from wave 1. In GPC C we repeat the strategy employed under (B) but restrict the sample to children who do not participate in a sports club in wave 1. The presented effect is the average treatment effect (ATE). p-values are computed *by bootstrapping p-values of the t-statistic with 4999 replications*.

	KiGGS		GCP A		GCP B		GCP C	
	Effect	p-val. %	Effect	p-val. %	Effect	p-val. %	Effect	p-val. %
**Health**								
Subjective Health	-0.08**	*2*	-0.12*	9	-0.10	*20*	0.02	*82*
**School performance**								
Overall Grade	-0.20***	*0*	-0.15**	*3*	-0.09	*11*	-0.19*	*7*
**Behavioural outcomes**								
Emotional P.	-0.09***	*0*	-0.08	*27*	-0.03	*59*	0.00	*98*
Behavioural P.	-0.02	*53*	-0.09	*12*	-0.07	*25*	-0.07	*52*
Hyperactivity	-0.01	*75*	0.08	*21*	0.07	*18*	0.20	*16*
Peer P.	-0.16***	*0*	-0.19***	*0*	-0.11**	*5*	-0.22**	*5*
Overall Score	-0.09***	*0*	-0.10*	9	-0.05	*32*	-0.02	*83*
Antisocial B.	-0.02	*59*	-0.02	*75*	-0.07	*22*	-0.06	*59*

*** denotes statistical significance at the 1% level, ** at the 5% level, and * at the 10% level.

When additionally including children's lagged outcome variables and replacing the control variables by control variables exclusively measured prior to treatment, the effects decrease slightly and accordingly loose statistical significance. The main picture, however, remains (see GCP B): children when participating in a sports club experience improvements in their health, school performance and behaviour. It is furthermore important to point out that children’s lagged outcome measures do not explain their active participation in sports clubs—the respective coefficients are insignificant in the propensity score estimation (see S2 Table, Column GCP C). Thus, prior health and human capital endowment is unlikely to explain selection into sports clubs.

The last column (GCP C) displays the estimates corrected for time constant unobserved heterogeneity by conditioning on sports-participation in the first wave of the panel. In so doing, we avoid that potentially endogenous control variables ‘mask’ some of the effects of sport participation. Moreover, by including the full set of lagged outcome variables we only compare children with the same initial skill endowment and health status. Our results are mostly robust to this correction: children's school performance improves by 0.19 sd (in comparison to 0.20 sd according to our baseline estimates), and children's peer problems reduce by 0.22 sd (in comparison to 0.16 sd according to our baseline estimates). Only the estimate of subjective health shrinks towards zero. In other words, sports club participation does not seem to influence children’s subjective health, at least not immediately (interviews are 1.5 years apart). Notice, however, that this result only sheds light on parents’ assessment of their children’s health. Unfortunately, we lack any objective health measures in the GCP to substantiate this finding.

Taken together, we are confident that unobserved time constant heterogeneity does not affect our results regarding children’s school performance and behaviour. Yet, we cannot claim the robustness of our results regarding children’s health status. Moreover, we cannot exclude the possibility that recent unobserved shocks may bias our results, both in terms of children’s health, education and behaviour. Nevertheless, the sensitivity analysis based on the simulation-based approach by Ichino, Mealli & Nannicini [32] reveals that our estimates robust with respect to confounders that are in the range of what might be expected for missing confounding variables.

### 5.2 Effect Heterogeneity

An important question from a policy perspective is whether the "right children" participate in sports, meaning whether those children who participate are those who benefit. We address this question in two different ways. On the one hand, we ask whether there is selection-on-gains—in other words, whether children who are participating in sports clubs are actually benefitting more from participation than children who are not participating. On the other hand, we analyze effect heterogeneity across children that differ in their observable characteristics.

To address the issue of selection-on-gains, we discuss, in addition to the average treatment effect (ATE), the average treatment effect on the treated (ATET) and the average treatment effect on the non-treated (ATENT). The ATET refers to the effect of sports participation on children who do engage in sports, while the ATENT refers to the effects on children, who do not participate, if they actually would participate (see Table 7). While for overall grades the ATENT is somewhat larger, the ATET is larger for health and behaviour. However, the overall picture remains mixed and the effects are not statistically different from each other at any conventional level. Thus, it does not seem that any of the two types of children would benefit significantly more from sports participation than the other type of children.

**Table 7:** Average effects for participants and nonparticipants (KiGGS). Note: p-values are computed by bootstrapping p-values of the t-statistic with 4999 replications. All variables are standardized to mean zero and variance one.

	Participants	p-val. %	Nonparticipants	p-val. %
**Health**				
Subjective Health	-0.15**	*1*	-0.09**	*3*
BMI	-0.03	*49*	0.01	*84*
Skinfold	-0.08**	*4*	-0.03	*44*
Puls	-0.16***	*0*	-0.08*	*7*
**School Perfomance**				
Overall Grade	-0.09	24	-0.20***	0
**Behavioural outcomes**				
Emotional Problems	-0.17***	*0*	-0.01	*78*
Behavioural Problems	-0.08	*11*	0.00	*95*
Hyperactivtiy	-0.07	*18*	0.00	*100*
Peer Problems	-0.26***	*0*	-0.17***	*0*
Overall Score	-0.19***	*0*	-0.05	*14*
Antisocial Behaviour	-0.05	*28*	-0.02	*58*

*** denotes statistical significance at the 1% level, ** at the 5% level, and * at the 10% level.

Table 8 presents effect heterogeneities with respect to observable characteristics. It contains the pair wise comparison of boys and girls, younger and older children, and finally children who live in cities and children who live in the countryside. Notice that due to a limited sample size when stratifying, we again abstain from controlling for state fixed effects. Thus, for the purpose of comparison with the estimates using the complete sample, please refer to the ones shown in Table 5, Column A.

**Table 8:** Heterogeneity with respect to other characteristics. Note: The distinction between city and countryside is based on INKAR and is a combination of population size, density, political and administrative relevance, etc. The presented effect is the average treatment effect (ATE). P-values are computed by bootstrapping p-values of the t-statistic with 4999 replications.

	Average Outcome		Avg.	p-val.	Average Outcome		Avg.	p-val.
	Part.	Not Par.	Effect	%	Part.	Not Part.	Effect	%
	**Panel A**							
	**City**				**Countryside**			
**Health**								
Subjective Health	-0.08	0.10	-0.18***	*0*	0.00	0.03	-0.03	*50*
BMI	-0.01	0.02	-0.04	*42*	0.02	0.04	-0.02	*65*
Skinfold	-0.03	0.07	-0.10*	*5*	0.00	0.07	-0.07	*15*
Puls	-0.06	0.04	-0.09***	*3*	-0.08	0.07	-0.15***	*0*
**School performance**								
Overall Grade	-0.03	0.13	-0.16	*18*	-0.1	0.03	-0.13	*11*
**Behavioural outcomes**								
Emotional Problems	-0.05	0.14	-0.19***	*0*	-0.02	-0.01	-0.01	*82*
Behavioural Problems	-0.01	0.06	-0.07	*20*	-0.02	-0.03	0.00	*94*
Hyperactivity	-0.04	0.04	-0.08*	*8*	0.00	-0.04	0.03	*39*
Peer Problems	-0.10	0.12	-0.22***	*0*	-0.09	0.05	-0.13***	*0*
**Overall Score**	**-0.06**	**0.12**	-0.19***	***0***	**-0.04**	**-0.02**	**-0.03**	***48***
Antisocial Behaviour	-0.04	-0.05	0.01	*80*	0.01	0.04	-0.03	*55*
	**Panel B**							
	**Boys**				**Girls**			
**Health**								
Subjective Health	-0.02	0.13	-0.16***	*0*	-0.05	-0.06	0.00	*96*
BMI	0.05	0.07	-0.02	*77*	-0.03	0.01	-0.03	*40*
Skinfold	-0.12	0.00	-0.11	*10*	0.09	0.15	-0.06	*15*
Puls	-0.14	-0.02	-0.11**	*1*	0.04	0.15	-0.11**	*1*
**School performance**								
Overall Grade	-0.02	0.12	-0.14	*25*	-0.14	0.11	-0.25***	*0*
**Behavioural outcomes**								
Emotional Problems	-0.07	-0.01	-0.06	*27*	-0.01	0.09	-0.1**	*3*
Behavioural Problems	0.08	0.13	-0.05**	*4*	-0.15	-0.09	-0.06	*15*
Hyperactivity	0.09	0.15	-0.05	*29*	-0.12	-0.15	0.03	*38*
Peer Problems	0.04	0.21	-0.17***	*0*	-0.22	0.03	-0.25***	*0*
**Overall Score**	**0.06**	**0.17**	-0.11**	***1***	**-0.17**	**-0.05**	-0.11***	***0***
Antisocial Behaviour	0.19	0.17	0.02	*83*	-0.25	-0.14	-0.11***	*0*
	**Panel C**							
	**Young (3–6 years)**				**Old (7–10 years)**			
**Health**								
Subjective Health	-0.08	0	-0.09**	*4*	-0.03	0.08	-0.11**	*2*
BMI	-0.32	-0.33	0.02	*59*	0.28	0.34	-0.05	*31*
Skinfold	-0.26	-0.23	-0.02	*53*	0.19	0.30	-0.11**	*3*
Puls	0.43	0.50	-0.07*	*9*	-0.48	-0.32	-0.16***	*0*
**School performance**								
Overall Grade	n.a.	n.a.	n.a.	n.a.	-0.08	0.12	-0.2***	*0*
**Behavioural outcomes**								
Emotional Problems	-0.12	-0.06	-0.06	*11*	0.05	0.18	-0.13**	*1*
Behavioural Problems	0.06	0.03	0.03	*48*	-0.09	0.02	-0.11**	*3*
Hyperactivity	-0.01	-0.02	0.00	*93*	-0.03	0.07	-0.10	*15*
Peer Problems	-0.13	0.02	-0.15***	*0*	-0.07	0.19	-0.26***	*0*
**Overall Score**	**-0.07**	**-0.01**	**-0.05**	***15***	**-0.04**	**0.16**	-0.20***	***0***
Antisocial Behaviour	0.11	0.07	0.04	*47*	-0.15	-0.04	-0.11*	*6*

*** denotes statistical significance at the 1% level, ** at the 5% level, and * at the 10% level.

The strongest differences exist when comparing children living in a city with children living on the countryside. "City" children who engage in a sports club experience a remarkable improvement in their subjective health (-0.18 sd) as well as in their objective health measures, such as skinfold (-0.10 sd) and pulse (-0.09). “Country” children do not gain much in terms of subjective health, but fare also better in terms of objective health measures (skinfold is reduced by -0.07 sd and pulse by -0.15 sd). Moreover, “city” children who participate in a sports club fare significantly better in terms of their behaviour (-0.19 sd). This improvement is mainly driven by a reduction in peer problems, emotional problems, and hyperactivity.

Interestingly, we also observe improved peer relations among "countryside" children when engaging in a sports club, yet no gain in any other dimension of behaviour. The underlying reason for the heterogeneous effects with respect to the degree of urbanization may be the respective counterfactual. While children living in a city might find it rather difficult to be physically active—the reason being simply a lack of outdoor space—children living on the countryside might be more physically active in general and thus, have a relatively lower gain from participating in a sports club than children living in a city. In fact, when using the country sample only, we do not observe a significant crowding out of sports outside a club due to sports in a club. However, the available measure may not necessarily capture general physical activity, such as running around or playing outside, and thus does not provide us with supportive empirical evidence for the statement made above.

Although girls generally score much better than boys on most of human capital indicators, sports club participation seems to equally affect boys and girls, with the exception of a slightly stronger effect on girls' anti-social behaviour. While we detect some heterogeneity in the effects on subjective health, these differences are not visible in the objective health measures. Yet, notice that previous literature also found only little evidence on heterogeneity in the treatment effects of sports participation [21].

When analysing the impact of sports club participation on the health and behaviour of younger and older children, we observe a slightly stronger effect for older children, the difference is, however, not significant.

### 5.3 Underlying mechanisms

One further important question from a policy perspective is what kind of activities children are actually exposed to when participating in a sports club and which kind of activities children are”sacrificing” when doing so. In other words, which are the underlying mechanisms through which sports club participation may exert its effects on children’s health, education and behaviour. In what follows, we first discuss the variety of activities encompassed by sports club participation. This discussion is based on the existing literature in developmental psychology. We then shed some light on the activities crowded out by sports club participation. For this purpose, we do not only rely on the existing literature but also investigate the impact of sports club participation on alternative activities undertaken by children empirically.

The obvious activity involved in sports club participation is physical exercise. Yet, participation in a sports club makes children go through a vast array of further activities and experiences that boost their development. According to Larson [55] and Hansen, Larson & Dworking [56] taking part in sport clubs may help children develop initiative, defined as an intrinsic motivation to plan, carry through, and achieve a valued goal. As such, participation in sports clubs, similar to participation in many other structured extracurricular activities, conveys “managerial” skills such as curiosity, effort and perseverance, responsibility, self-assessment, as well as project, time and stress management. In addition, the interaction with a team and in particular the interaction with peers who would normally be outside the existing social network, fosters the development of personal skills such as empathy, loyalty, intimacy, self-control, team-spirit, but also dealing with criticism and conflict.

The additional advantage of exercising sports in a club in contrast to pure physical exercise is the fact that it is a guided and highly structured extra-curricular activity [57,58]. It not only imposes structure on children’s life but also puts children in contact with instructors and competent peers that may act as role models. As such, sports club participation may foster children’s development also indirectly through reducing time in unstructured, less beneficial, activities. The available data allow us to investigate this channel empirically. Table 9 shows the impact of sports club participation on the time spent in physical activities undertaken outside a sports club and passive activities such as watching TV and using a computer.

**Table 9:** Average effects on alternative activities (KiGGS). Note: P-values are computed by bootstrapping p-values of the t-statistic with 4999 replications. Sports exercised outside a club is measured as a binary variable, where 1 indicates a child is doing at least once per week sports outside a club. All other activities are measured as hours per day.

	Participants	Nonparticipants	Avg. Effect	p-val.%
**Physical activity outside of a sports club**				
Sports outside a club	0.53	*0*.*52*	0.01	*63*
**Passive activities**				
Watching TV on a week day	0.99	1.06	-0.07***	*0*
Watching TV on weekend	1.61	1.68	-0.06**	*4*
Using PC on a week day	0.21	0.20	0.01	*46*
Using PC on the weekend	0.44	0.42	0.02	*23*

*** denotes statistical significance at the 1% level and ** at the 5% level.

Interestingly, the reported level of sports activities done *outside* a club is the same among children who participate in a sports club and among children who do not. Given this result, it seems safe to say that sports club participation stimulates children’s overall physical activity and is not just a substitute for other sports activities. Yet, perhaps more interestingly, our results provide evidence that sports club participation leads to a small, but significant crowding out of TV consumption by 4.2 minutes on a weekday and 3.6 minutes on Saturdays and Sundays. Putting this finding into relation with the average attendance of children at a sports club, we can infer that exercising approximately 1–2 times per week in a sports club leads to a reduction of 28 minutes TV watching per week. It seems unlikely that this finding can fully explain the substantial improvements in children’s human capital due to sports club participation. Unfortunately, KiGGS does not provide us with any further information about children’s leisure activities nor about their time devoted to school-related activities, such as homework. Therefore, we may conclude that sports club participation crowds out some “passive” activities, but we do not know whether it stimulates further “active” or “development stimulating” activities.

## 6 Conclusion

While different disciplines acknowledge the importance of acquiring good health, education and behaviour during childhood for outcomes later in life, the role of extra-curricular activities for their formation is not yet fully understood. For this purpose, we investigate the effects of sports activities on different measures of health, school performance and behaviour among preschool and primary school age children.

Our results indicate positive effects of participation in sports on children's health, education and behaviour: overall, children's school grades and behaviour, in particular the relation to their peers, improve substantially; Results are robust when using different data and empirical strategies as well as when including simulated unobserved confounding variables. Only initial beneficial effects on children’s health are not robust to the inclusion of prior health conditions.

Our results highlight the importance of physical activities for children's development. Encouraging children to participate in sports and providing the necessary infrastructure should therefore be, and in many countries already is, an important policy objective. Further research should qualify this statement by a cost-benefit analysis.

Our results provide also evidence that the positive effects of doing sports in a club are partially explained by an increase in physical activity as sports club participation does not crowd out other sports activities. The effects are strongest in cities, where children have fewer opportunities to be physically active outside of sports clubs—as well as by a reduction in passive activities such as watching TV. Nevertheless, "doing sports in a club" has still many more dimensions, which, given the data at hand, we are not able to explore. Participating in a sports club challenges children to take initiative and to plan, carry through, and achieve a valued goal. Sports club participation exposes children to cooperation with other children in a team, which may make them better *team players* also in other situations in life and, thus, may explain the reduction in peer problems. Doing sports in a club comes often along with participation in competitions. Victory in competition may raise children's self-esteem while defeat, despite eventual negative effects on children’s self-esteem, may teach them how to deal with such a situation. Future research should therefore try to dig deeper into the mechanisms through which sports activities may influence skill formation and disentangle the various channels through which the effect may work.

While providing evidence on the short-run effects of sports club participation, our study falls short in assessing its long term or sustained effects into young adulthood and beyond. In addition, our study does not answer whether sustained effects depend on continued participation in sports clubs or an equivalent exercise regimen in later years. Such questions are important to assess the overall benefit of sports clubs and should be addressed in future research.

## Supporting Information

S1 Appendix Further details on the estimator used—Matching. (DOCX)

S2 Appendix Further details on the estimator used—Semi-parametric IV. (DOCX)

S3 Appendix Further details on the estimator used—Formal sensitivity analysis of matching estimation. (DOCX)

S4 Appendix Non-cognitive skills—The Strength and Difficulties Questionnaire (SDQ). (DOCX)

S5 Appendix Construction of the non-cognitive skills using the SDQ. (DOCX)

S6 Appendix Well-being—The KINDL-R Questionnaire. (DOCX)

S1 Table Descriptive Statistics of the control variables and coefficients of the propensity score estimation based on the KiGGS sample (probit). (DOCX)

S2 Table Further Estimation results—Propensity score estimation (probit) using the GCP dataset. (DOCX)

S3 Table Further Estimation results—Propensity score estimation (probit) for the semi-parametric LATE. (DOCX)

S4 Table Additional Estimates—Comparison of matching estimates for well-being using KiGGS and GCP. (DOCX)

S5 Table Additional Estimates—Descriptive statistics for children's cognitive and non-cognitive skills (non-standardized variables). (DOCX)

S6 Table Additional Estimates—Matching estimates for cognitive and non-cognitive skills (KiGGS) (non-standardized variables). (DOCX)

S7 Table Additional Estimates—Matching estimates for cognitive and non-cognitive skills using different propensity score specifications (KiGGS). (DOCX)

S8 Table Additional Estimates—Matching estimates for non-cognitive skills (KiGGS—cognitive sample). (DOCX)

S9 Table Additional Estimates—Bootstrapped difference in average treatment effects between specifications with and without state fixed effects. (DOCX)

S10 Table Balancing tests—After-match balancing tests (KiGGS). (DOCX)

S11 Table Balancing tests—After-match balancing tests (GCP). (DOCX)

S12 Table Balancing tests—After-match balance tests for semi-parametric IV. (DOCX)

## References

[1] Murnane R., Willett J., & Levy F. (1995). The Growing Importance of Cognitive Skills in Wage Determination. The Review of Economics and Statistics, 77 (2): 251–266.

[2] Cawley J., Heckman J., Lochner L., & Vytlacil E. (2000). Understanding the Role of Cognitive Ability in Accounting for the Recent Rise in the Economic Return to Education In Arrow K., Bowles S., & Durlauf S., Meritocracy and Economic Inequality (pp. 230–265). Princeton: Princeton University Press.

[3] Heckman J., Stixrud J., & Urzua E. (2006). The Effects of Cognitive and Noncognitive Abilities on Labour Market Outcomes and Social Behaviour. Journal of Labour Economics, 24 (3): 411–482.

[4] Currie J., & Bridget M. (1999). Health, health insurance, and the labour market In Ashenfelter O., & Card D., Handbook of Labour Economics (S. Amsterdam: North Holland).

[5] Smith J. (1999). Healthy Bodies and Thick Wallets: The Dual Relation between Health and Economic Status. Journal of Economic Perspectives, 13 (2): 145–166. PMC3697076 15179962

[6] Grossman M. (2000). The Human Capital Model In Culyer A., & Newhouse J., The Handbook of Health Economics. Amsterdam: North Holland.

[7] Case A., Fertig A., & Paxson C. (2005). The lasting impact of childhood health. Journal of Health Economics, 24 (2): 365–389. 15721050 10.1016/j.jhealeco.2004.09.008

[8] Currie J. (2009). Healthy, Wealthy, and Wise: Socioeconomic Status, Poor Health in Childhood, and Human Capital Development. Journal of Economic Literature, 47 (1): 87–122.

[9] Gluckmann P., & Hanson M. (2005). The Fetal Matrix: Evolution, Development and Disease. Cambridge, UK: Cambridge University Press.

[10] Heckman J. (2007). The technology and neuroscience of capacity formation. Proceedings of the National Academy of Science, 104(33): 13250–13255. 10.1073/pnas.0701362104 PMC1948899 17686985

[11] Currie J., & Almond D. (2011). Human Capital Development before Age Five. In Ashenfelter O., & Card D., Handbook of Labour Economics (Vol. 4(2): 1315–1486).

[12] Kutteroff A., & Behrens P. (2006). *KIM Studie 2006*: *Kinder und Medien*, *Computer und Internet*. Medienpädagogischer Forschungsverbund Südwest.

[13] Strong W., Malina R., Blimkie C., Daniels S., Dishman R., Gutin B, et al (2005). Evidence Based Physical Activity for School-Age Youth. The Journal of Pediatrics, 146 (6): 732–737. 15973308 10.1016/j.jpeds.2005.01.055

[14] Morrow J. R., Tucker J. S., Jackson A. W., Martin S. B., Greenleaf C. A., & Petrie T. A. (2013). Meeting Physical Activity Guidelines and Health-related Fitness in Youth. *American Journal of Preventive Medicine*, 44 (5): 439–444. 10.1016/j.amepre.2013.01.008 23597805

[15] Meyer U., Romann M., Zahner L., Schindler C., Puder J. J., Kraenzlin M. et al (2011). Effect of a general school-based physical activity intervention on bone mineral content and density: a cluster-randomized controlled trial. Bone, 48 (4): 792–797. 10.1016/j.bone.2010.11.018 21167330

[16] Dills A., Morgan H., & Rotthoff K. (2011). “Recess, Physical Education, and Elementary School Student Outcomes. Economics of Education Review, 30(5): 889–900.

[17] Barron J., Ewing B., & Waddell G. (2000). The Effects of High School Athletic Particpiation on Education and Labour Market outcomes. Review of Economics and Statistics, 82 (3): 409–421.

[18] Eccles J., Barber B., Stone M., & Hunt J. (2003). Extracurricular activities and adolescent development. Journal of social issues, 59(4): 865–889.

[19] Eide E., & Ronan N. (2001). Is participation in high school athletics an investment or a consumption good? Evidence from high school and beyond. Economics of Education Review, 20 (5): 431–442.

[20] Pfeifer C., & Cornelissen T. (2010). The impact of participation in sports on educational attainment—New evidence from Germany. Economics of Education Review, 29 (1): 94–103.

[21] Stevenson B. (2010). Beyond the Classroom: Using Title IX to Measure the Return to High School Sports. Review of Economics and Statistics, 92(2): 284–301.

[22] Rees D., & Sabia J. (2010). Sports participation and academic performance: Evidence from the National Longitudinal Study of Adolescent Health. Economics of Education Review, 29 (5): 751–759.

[23] Cunha F., Heckman J., Lochner L., & Masterov D. (2006). Interpreting the Evidence on Life Cycle Skill Formation In Hanushek E., & Welch F., Handbook of the Economics of Education (pp. 698–812). Elsevier B.V.

[24] Bronfenbrenner U. (1986). Ecology of the family as a context for human development: Research perspectives. Developmental Psychology, 22(6): 723–742.

[25] Cawley J., Frisvold D., & Meyerhofer C. (2013). The Impact of Physical Education on Obesity among Elementary School Children. Journal of Health Economics, S. 18341. 10.1016/j.jhealeco.2013.04.006 23721885

[26] Tietjens M. (2001). *Sportliches Engagement und sozialer Rückhalt im Jugendalter* Eine repräsentative Surveystudie in Brandenburg und Nordrhein-Westfalen. Lengerich: Pabst.

[27] Kurth B., Kamtsiuris P., Hölling H., Schlaud M., Dölle R., Ellert U. et al (2008). The challenge of comprehensively mapping children's health in a nation-wide health survey: Design of the German KiGGS-Study. BMC Public health, 8: 196 10.1186/1471-2458-8-196 18533019 PMC2442072

[28] Goodman R. (1997). The Strengths and Difficulties Questionnaire: A Research Note. Journal of Child Psychology and Psychiatry, 38 (5): 581–586. 9255702 10.1111/j.1469-7610.1997.tb01545.x

[29] Muris P., Meesters C., & van den Berg F. (2003). The Strengths and Difficulties Questionnaire (SDQ): Further evidence for its reliability and validity in a community sample of Dutch children and adolescents. European Child and Adolescent Psychiatry, 12 (1): 1–8. 12601558 10.1007/s00787-003-0298-2

[30] Goodman R., Meltzer H., & Bailey V. (1998). The Strengths and Difficulties Questionnaire: A pilot study on the validity of the self-report version. European Child and Adolescent Psychiatry, 7 (3): 125–130. 9826298 10.1007/s007870050057

[31] Gallagher. (1996). How useful is BMI for comparison of body fatness across age, sex and ethnic groups? American Journal of Epidemiology, 143 (3): 228–239. 8561156 10.1093/oxfordjournals.aje.a008733

[32] Stattin H. & Kerr M. (2009). Neighborhood contexts of peer relationships and groups In Rubin K. H., Bukowski W., & Laursen B. (Eds.). Handbook of peer relationships. Peer interactions and groups, Ch. 23: 414–431. New York: Wiley.

[33] Ichino A., Mealli F., & Nannicini T. (2008). From Temporary Help Jobs to Permanent Employment: What Can We Learn from Matching Estimators and their Sensitivity? Journal of Applied Econometrics, 23: 305–32.

[34] Rosenbaum P., & Rubin D. (1983). Assessing sensitivity to an unobserved binary covariate in an observational study with binary outcome. Journal of the Royal Statistical Society—Series B, 45: 212–218.

[35] Leibowitz A. (1974). Home investments in children. Journal of Political Economy, 82 (2): 111–131.

[36] Blau D. (1999). The Effect of Income on Child Development. The Review of Economic and Statistics, 81 (2): 261–276.

[37] Case A., Lubotsky D., & Paxson C. (2002). Economic Status and Health in Childhood: The Origins of the Gradient. American Economic Review, 92 (5), pp. 1308–1334. 29058397 10.1257/000282802762024520

[38] Black S., Devereux P., & Salvanes K. (2005). Why the Apple doesn’t fall far: Understanding Intergenerational Transmission of Human Capital. The American Economic Review, 95 (1): 437–449.

[39] Kling J., Liebman J., & Katz L. (2007). Experimental Analysis of Neighborhood Effects. Econometrica, 75 (1): 83–119.

[40] Williams W., & Sternberg R. (2002). How parents can maximize children’s cognitive competence In Bornstein M., *Handbook of Parenting* (2nd ed., pp. 169–194). Mahwah, New Jersey: Lawrence Erlbaum Associates.

[41] Persico N., Postlewaite A., & Silverman D. (2004). The Effect of Adolescent Experience on Labour Market Outcomes: The Case of Height. Journal of Political Economy, 112 (5): 1019–1053.

[42] Lechner M. & Wunsch C. (2013). Sensitivity of Matching-based Program Evaluations to the Availability of Control Variables, Labour Economics, 21(C), 111–121.

[43] Lechner M. (2009). Long-Run Labour Market Effects of Individual Sports Activities. Journal of Health Economics, 28 (4): 839–854. 10.1016/j.jhealeco.2009.05.003 19570587

[44] Nannincini T. (2007). Simulation-based Sensitivity Analysis for Matching Estimators. Stata Journal, 7 (3), 334–350.

[45] Altonji J., Elder T., & Taber C. (2005). Selection on observed and unobserved variables: Assessing the effectiveness of Catholic Schools. Journal of Political Economy, 113: 151–184.

[46] Rosenbaum P. (1997). Sensitivity analysis to certain permutation inferences in matched observational studies. Biometrika, 74: 13–26.

[47] Imbens G. (2003). Sensitivity to exogeneity assumptions in program evaluation. American Economic Review: Papers and Proceedings, 93: 126–132.

[48] Steinmayr A., Felfe C., & Lechner M. (2011). The closer the sportier? Children's sport activity and their distance to sport facilities. European Review of Ageing and Physical Activity, 8 (2): 67–82.

[49] Felfe C., Lechner M., & Steinmayr A. (2011). Sport and Child Development. CESifo Working Papers, 3629.

[50] Lechner M., Miquel R., & Wunsch C. (2011). Long-Run Effects of Public Sector Sponsored Training in West Germany. Journal of the European Economic Association, 9 (4): 742–784.

[51] Huber M., Lechner M., & Wunsch C. (2013). The Performance of Estimators Based on the Propensity Score. Journal of Econometrics, 175 (1): 1–21.

[52] Huber M., Lechner M., & Steinmayr A. (2014). Radius Matching on the Propensity Score with Bias Adjustment: Finite Sample Behaviour, Tuning Parameters and Software Implementation. Empirical Economics, 49 (1): 1–31.

[53] Rosenbaum P., & Rubin D. (1983). The Central Role of the Propensity Score in Observational Studies for Causal Effects. Biometrika, 70 (1): 41–55.

[54] Felfe C., & Lalive R. (2014). Does Early Childcare Help or Hurt Child Development?. IZA DP 8400.

[55] Larson R. (2000). Toward a psychology of positive youth development. American Psychologist;55(1): 170–83 11392861 10.1037//0003-066x.55.1.170

[56] Hansen D., Larson R., & Dworkin J. (2003). What Adolescents Learn in Organized Youth Activities: A Survey of Self-Reported Developmental Experiences. Journal of Research on Adolescence, 13(1): 25–55

[57] Mahoney J. L., & Cairns R. B. (2000). Extracurricular activities In Craig W. (Ed.), Childhood social development: The essential readings (pp. 167–196). Oxford, U.K: Blackwell Publishers, Inc.

[58] Mahoney J. L., Larson R., Eccles J. S., & *Lord H. (2005). Organized activities as developmental contexts for children and adolescents In Mahoney J. L., Larson R. W., & Eccles J. S. (Eds.), Organized activities as contexts of development: Extracurricular activities, after-school and community programs (pp. 3–22). Mahwah, NJ: Lawrence Erlbaum & Associates

