# Supplementary material for: Sports and Child Development
Source: PLoS One. 2016 May 4;11(5):e0151729. doi: 10.1371/journal.pone.0151729 (PMC4856309; doi:10.1371/journal.pone.0151729)
Supplement: S1 Appendix — (DOCX) [file pone.0151729.s001.docx]

# S1 Appendix: Further details on the estimator used - Matching

This appendix describes the baseline matching protocol used for the match­ing and the instrumental variable estimators.

S1 Table 1: A matching protocol for the estimation of a counterfactual outcome and the effects

| Step A-1 | Choose one observation in the subsample defined by treatment *d*=1 and delete it from that pool. |
| --- | --- |
| Step B-1 | Find an observation in the subsample defined by *d*=0 that is as close as possible to the one chosen in step A-1) in terms of $p(x),\tilde{x}$. 'Closeness' is based on the Mahalanobis dis­tance.  |
| Step C-1 | Repeat A-1) and B-1) until no observation with *d*=1 is left. |
| Step D-1 | Compute the distribution of distances obtained for any comparison between a member of the reference distri­bution and matched comparison observations. Obtain the distance at quantile *Q* (*dist*). |
| Step A-2 | Repeat A-1). |
| Step B-2 | Repeat B-1). If possible, find other observations in the subsample of *d*=0 that are at least as close as *R _*_ dist* to the one chosen in step A-2). Do not remove these observations, so that they can be used again. Compute weights for all chosen comparisons observations that are pro­portional to their distance. Normalize the weights such that they add to one. |
| Step C-2 | Repeat A-2) and B-2) until no participant in *d*=1 is left. |
| Step D-2 | D-2) For any potential comparison observation, add the weights obtained in A-2) and B-2). |
| Step E | Using the weights $w(x_{i})$ obtained in D-2), run a weighted linear regression of the outcome variable on the variables used to define the distance (and an intercept). |
| Step F-1 | Predict the potential outcome $y^{0}(x_{i})$ of every observation using the coefficients of this regres­sion: $\hat{y}^{0}(x_{i})$.  |
| Step F-2 | Estimate the bias of the matching estimator for $E(Y^{0}\vert D=1)$ as: .  |
| Step G | Using the weights obtained by weighted matching in D-2), compute a weighted mean of the outcome variables in *d=0*. Subtract the bias from this estimate to get $\widehat{E(Y^{0}\vert D=1)}$.  |

Note: *R* is set to 90%, Q is set to 90..

The parameters used to define the radius for the distance-weighted radius matching are set to 0.9 for both, *R* and Q. This value refers to the distance of the worst match in a one-to-one matching and is defined in terms of the propensity score. Differ­ent values for *R* and *Q* are checked in the sensitivity analysis in Lechner, Miquel, and Wunsch (2011) as well as in the simulation study by Hu­ber, Lechner, and Wunsch (2010). They showed a considerable robustness of the results with re­spect to the choice of *R* and *Q*.
