# Supplementary material for: Sports and Child Development
Source: PLoS One. 2016 May 4;11(5):e0151729. doi: 10.1371/journal.pone.0151729 (PMC4856309; doi:10.1371/journal.pone.0151729)
Supplement: S2 Appendix — (DOCX) [file pone.0151729.s002.docx]

# S2 Appendix: Further details on the estimator used – Semi-parametric IV

This appendix gives a more technical introduction into the estimator used for the semi-parametric LATE:

We use procedures that take the form of a ratio of two propensity score matching estimators. Such procedures were proposed by Frölich (2007). Under our maintained assumption of effect heterogeneity, the LATE is the causal effect of a change in the distance for the subpopulation of individuals who would react to such a change with a change in sports activity status.

It is an extension to the framework of Imbens and Angrist (1994) who dis­cuss identification for an unconditionally valid instrument to the case when the instrument $Z$ is only valid conditional on observed factors $X$. For simplic­ity, assume the instrument, i.e. the distance to sports facility would be binary, e.g. a long versus a short distance. Let $p_{Z}(x)$ denote $\Pr(Z=1|X=x)$, the condi­tional probability of short distance given the observed factors. The LATE is given by

where $Y$ is the child's outcome, $D$ denotes the sports state, and $f_{p_{_{Z}}(X)}(p_{Z}(x))$denotes the density of $p_{Z}(X)$.

Thus, the LATE can be estimated by (i) matching the outcomes $Y$ in the sub­groups $Z=1$ and $Z=0$on the estimated $p_{Z}(X)$, (ii) matching the employ­ment states $D$ in the subgroups $Z=1$ and $Z=0$ on the estimated $p_{Z}(X)$, and (iii) computing the ratio of the former to the latter. Frölich (2007) shows that this method is consistent and asymptotically normal under standard regularity condi­tions. Here, $p_{Z}(X)$ is estimated by a probit regression (the specification and coeffi­cient estimates are provided in Appendix C). For either matching step, we use the same methods as for estimation under the CIA. Note that the matching procedure for the numerator yields the estimate for the intention to treat effect (ITT), while the one for the denominator estimates the proportion of the com­pliers. Thus, the LATE is equal to the ITT inflated by the share of compliers. While identification is only feasible if compliers exist, estimation is only precise if the share of com­pliers is not too small, implying that the instrument is suffi­ciently relevant.
