# Supplementary material for: Sports and Child Development
Source: PLoS One. 2016 May 4;11(5):e0151729. doi: 10.1371/journal.pone.0151729 (PMC4856309; doi:10.1371/journal.pone.0151729)
Supplement: S3 Appendix — (DOCX) [file pone.0151729.s003.docx]

# S3 Appendix: Further details on the estimator used – Formal sensitivity analysis of matching estimation

In this section, the details of the sensitivity analysis based on the flexible approach of Ichino, Mealli, and Nannicini (2008) and Nannicini (2007) are discussed. The idea is to simulate a confounder that is related to the potential out­comes and the treatment and to test whether its inclusion in the propensity score affects the results. Comparing the estimates with and without this new covariate indicates the sensitivity of the chosen matching specification to missing confounding variables. Alt­hough, the ap­proach is designed specifically for the estimation of the average treatment effect on the treated, the gen­eral principle can be applied to all mean treatment effects usually considered in the litera­ture. In turn we discuss how we implement this simulation based approach.

In order to simplify the simulations of the new unobserved confounder, both the unobserved confounder as well as the outcomes are treated as binary and independent of (included) covariates. The key design parameters for simulating the new unobserved confounder, *U*, are its probability for taking a value of 1 that varies for the four strata defined by a binary outcome and a binary treatment, i.e.$p^{ij}=P(U=1|Y=i,D=j), i,j\in\{0,1\}$ (*Y* denotes the outcome variable and *D* denotes the treatment). If the values of $p^{ij}$ vary with treatment and outcome, then including *U* among the confounders has the potential to affect the results even after controlling for all covari­ates in the model. We choose the design probabilities such that they reflect i) an artificial unbalanced design which is expected to lead to poten­tially substantial biases and ii) the correlation patterns of important observed confounders. The latter attempts to answer the question of how much bias would be there if there are other confounding variables omitted that share the same ‘con­founding struc­ture/potential’ as those important control variables, but are at the same time in­dependent of those and all other already included controls.

Implementation in our setting requires several further deci­sions. Many of those decisions have also the purpose of keeping the complexity, and thus the computational costs, at bay. The first decision relates to the outcome variable chosen for the simulation of the new unobserved confounder. We chose as outcome variable subjective health and split it at the median category (in other words we create a dummy variable indicating whether an individual is in good or bad health; this is done for the purpose of simulation of the artificial confounders only). The reason for choosing subjective health is that it is also a key driver for all other outcome variables. The second decision is on how to choose the values of $p^{ij}$. We consider four scenarios: The first one is a ‘neu­tral’ scenario with $p^{ij}=0.5, \forall i,j$. In this case including the simulated confounder should not lead to any bias and thus provides a benchmark which allows us to check whether the ran­domness induced by the sim­ulation has any impact. We can then compare these benchmark estimates to more selective scenarios. Three further scenarios mimic the relation of the outcome variable and the treat­ment to three important confounders (we choose the income of the family, the education of the mother, and the regional employment rate). Thus, in those scenarios the $p^{ij}$’s vary according to their empirical distribution in the sample as shown in S2 Table 2.

S3 Table 1: Correlation structure of artificial confounders (in %)

|  | $P(U=1\vert D=1,Y=1)$ | $P(U=1\vert D=1,Y=0)$ | $P(U=1\vert D=0,Y=1)$ | $P(U=1\vert D=0,Y=0)$ |
| --- | --- | --- | --- | --- |
| Income > 5000 EUR | 5.5 | 6.3 | 1.4 | 2.6 |
| Mom’s education: university | 0.9 | 0.8 | 6.3 | 3.4 |
| Regional employment rate | 43 | 49 | 52 | 48 |
| Baseline | 50 | 50 | 50 | 50 |

Since for each observation ‘*i*’ the realisation of $u_{i}$ will be random, we fol­low the suggestion of Ichino, Mealli, and Nannicini (2008) to repeat the simulations many times and then take the mean of the effects over the simulations. The simulated confounding variables are then included in the propensity score and estimation proceeds in the standard way.

Finally, there is the issue of how to obtain inference in this case and of how many times to draw from the distribution of *U*. With respect to inference it appears natural to use boot­strap inference as the true asymptotics are not known for the matching estimator used. Since this procedure is computational very intensive in our rather large samples, we set the number of draws of *U* (within each bootstrap replication) to 19 and use only 99 bootstrap rep­lica­tions. The test statistics are based on the difference of the baseline scenario to the vari­ous con­founding scenarios using the quantile method. In Table 5, we report those devia­tions from the baseline results as well as their significance. In fact, none of deviations is significant at the 10% level.
