# Supplementary material for: Sports and Child Development
Source: PLoS One. 2016 May 4;11(5):e0151729. doi: 10.1371/journal.pone.0151729 (PMC4856309; doi:10.1371/journal.pone.0151729)
Supplement: S5 Appendix — (DOCX) [file pone.0151729.s005.docx]

# S5 Appendix: Construction of the non-cognitive skills using the SDQ
