# Supplementary material for: Sports and Child Development
Source: PLoS One. 2016 May 4;11(5):e0151729. doi: 10.1371/journal.pone.0151729 (PMC4856309; doi:10.1371/journal.pone.0151729)
Supplement: S1 Table — (DOCX) [file pone.0151729.s007.docx]

# S1 Table: Descriptive statistics of the control variables and coefficients of the propensity score estimation based on the KiGGS sample (probit)

|  | No Sports | | Sports | | Sports - No Sports | | | Probit Coefficient | |
| --- | --- | --- | --- | --- | --- | --- | --- | --- | --- |
|  |  |  | | Diff. | | p-val. % | Coef. | | p-val. % |
| **Child characteristics** |  |  | |  | |  |  | |  |
| Male | 0.50 | 0.52 | | 0.02 | | *13* | 0.06 | | *10* |
| Age: 3 years | 0.17 | 0.05 | | -0.11 | | *0* | -0.76 | | *0* |
| 4 years | 0.15 | 0.09 | | -0.07 | | *0* | -0.41 | | *0* |
| 5 years | 0.13 | 0.11 | | -0.02 | | *5* | -0.12 | | *11* |
| 6 years | 0.13 | 0.13 | | 0.01 | | *52* | 0.16 | | *3* |
| 7 years | 0.11 | 0.15 | | 0.04 | | *0* | 0.27 | | *0* |
| 8 years | 0.10 | 0.15 | | 0.05 | | *0* | 0.31 | | *0* |
| 9 years | 0.11 | 0.16 | | 0.06 | | *0* | 0.17 | | *11* |
| 10 years | 0.10 | 0.14 | | 0.04 | | *0* | -0.76 | | *0* |
| Height in cm | 121.0 | 127.6 | | 6.6 | | *0* | 0.00 | | 28 |
| Birthweight in grams | 3346.7 | 3361.6 | | 14.9 | | *40* | 0.00 | | 89 |
| **Mother's characteristics** |  |  | |  | |  |  | |  |
| Education: Basic | 0.20 | 0.15 | | -0.05 | | *0* | -0.14 | | 1 |
| Intermediate | 0.49 | 0.47 | | -0.03 | | *5* | ref. | |  |
| High school | 0.12 | 0.18 | | 0.06 | | *0* | 0.09 | | 11 |
| University | 0.14 | 0.19 | | 0.05 | | *0* | 0.02 | | 76 |
| Other | 0.04 | 0.01 | | -0.03 | | *0* | -0.61 | | 0 |
| LFP: Not working | 0.17 | 0.18 | | 0.01 | | *29* | -0.07 | | 21 |
| Unemployed | 0.14 | 0.06 | | -0.08 | | *0* | -0.12 | | 11 |
| Maternal leave | 0.10 | 0.10 | | -0.01 | | *24* | 0.01 | | 87 |
| Part time | 0.37 | 0.51 | | 0.14 | | *0* | ref. | |  |
| Fulltime | 0.20 | 0.15 | | -0.06 | | *0* | -0.17 | | 0 |
| Job: Unskilled | 0.24 | 0.19 | | -0.06 | | *0* | -0.18 | | 0 |
| Semiskilled | 0.45 | 0.53 | | 0.08 | | *0* | ref. | |  |
| Highskilled | 0.06 | 0.08 | | 0.02 | | *0* | -0.04 | | 62 |
| Self employed | 0.06 | 0.07 | | 0.01 | | *0* | -0.08 | | 34 |
| Other job | 0.03 | 0.01 | | -0.02 | | *0* | ref. | |  |
| Housewife | 0.13 | 0.11 | | -0.02 | | *0* | -0.09 | | 18 |
| BMI: Underweight | 0.03 | 0.03 | | 0.00 | | *79* | 0.02 | | 83 |
| Normal | 0.61 | 0.67 | | 0.06 | | *0* | ref. | |  |
| Overweight | 0.23 | 0.21 | | -0.02 | | *10* | -0.02 | | 69 |
| Obese | 0.12 | 0.09 | | -0.03 | | *0* | -0.06 | | 35 |

Note: S1 Table 1 to be continued.

S1 Table continued

|  | No Sports | | Sports | | Sports - No Sports | | | Probit Coefficient | |
| --- | --- | --- | --- | --- | --- | --- | --- | --- | --- |
|  |  |  | | Diff. | | p-val. % | Coef. | | p-val. % |
| **Father's characteristics** |  |  | |  | |  |  | |  |
| Education: Basic | 0.24 | 0.24 | | 0.00 | | *72* | 0.04 | | 44 |
| Intermediate | 0.40 | 0.32 | | -0.08 | | *0* | ref. | |  |
| High school | 0.08 | 0.12 | | 0.04 | | *0* | 0.13 | | 5 |
| University | 0.19 | 0.28 | | 0.10 | | *0* | -0.03 | | 71 |
| Other | 0.03 | 0.01 | | -0.02 | | *0* | 0.00 | | 100 |
| LFP: Not working | 0.02 | 0.02 | | 0.00 | | *91* | 0.22 | | 9 |
| Unemployed | 0.10 | 0.04 | | -0.07 | | *0* | -0.13 | | 11 |
| Paternal leave | 0.00 | 0.00 | | 0.00 | | *70* | ref. | |  |
| Parttime | 0.03 | 0.03 | | 0.00 | | *48* | -0.02 | | 88 |
| Fulltime | 0.79 | 0.89 | | 0.10 | | *0* | ref. | |  |
| Job: Unskilled | 0.14 | 0.08 | | -0.06 | | *0* | -0.13 | | 4 |
| Semiskilled | 0.51 | 0.47 | | -0.04 | | *0* | ref. | |  |
| Highskilled | 0.14 | 0.24 | | 0.11 | | *0* | 0.10 | | 10 |
| Self employed | 0.12 | 0.17 | | 0.04 | | *0* | 0.09 | | 13 |
| Other job | 0.01 | 0.01 | | 0.00 | | *82* | ref. | |  |
| Houseman | 0.01 | 0.00 | | -0.01 | | *0* | ref. | |  |
| BMI: Underweight | 0.00 | 0.00 | | 0.00 | | *50* | ref. | |  |
| Normal | 0.35 | 0.40 | | 0.05 | | *0* |  |  |  |
| Overweight | 0.38 | 0.43 | | 0.05 | | *0* | 0.04 | | 36 |
| Obese | 0.12 | 0.09 | | -0.03 | | *0* | -0.12 | | 7 |
| Missing | 0.15 | 0.09 | | -0.06 | | *0* | -0.26 | | 0 |
| **Family characteristics** |  |  | |  | |  |  | |  |
| Social class: Low | 0.31 | 0.15 | | -0.16 | | *0* | -0.16 | | 1 |
| Medium | 0.46 | 0.49 | | 0.03 | | *2* | ref. | |  |
| High | 0.22 | 0.36 | | 0.14 | | *0* | 0.01 | | 94 |
| Total household income | 2024.9 | 2336.1 | | 311.2 | | *0* | 0.00 | | 0 |
| > 5000 (binary) | 0.02 | 0.06 | | 0.04 | | *0* | 0.50 | | 0 |
| Missing (binary) | 0.04 | 0.04 | | 0.00 | | *78.5* | 0.28 | | 2 |
| Single parent household | 0.13 | 0.08 | | -0.04 | | *0* | 0.03 | | 69 |
| Siblings in household | 1.13 | 1.12 | | -0.01 | | *82* | -0.10 | | 0 |
| Older sibling in household (binary) | 0.50 | 0.49 | | -0.01 | | *47* | 0.02 | | 58 |
| Mold at home | 0.06 | 0.03 | | -0.02 | | *0* | -0.23 | | 1 |

Note: S1 Table 1 to be continued.

S1 Table continued

|  | No Sports | | Sports | | Sports - No Sports | | Probit Coefficient | | |
| --- | --- | --- | --- | --- | --- | --- | --- | --- | --- |
|  | Mean | Mean | | Diff. | | p-val. % | | Coef. | p-val. % |
| **Parenting style** |  |  | |  | |  | |  |  |
| Smoking during pregnancy: regularly | 0.06 | 0.03 | | -0.04 | | *0* | | -0.39 | 0 |
| occasionally | 0.15 | 0.11 | | -0.04 | | *0* | | -0.12 | 3 |
| never | 0.77 | 0.85 | | 0.08 | | *0* | | ref. |  |
| Family cares: no | 0.01 | 0.00 | | 0.00 | | *35* | | 0.05 | 72 |
| rather no | 0.02 | 0.02 | | 0.00 | | *21* | |  |  |
| rather yes | 0.40 | 0.43 | | 0.03 | | *1* | | 0.11 | 1 |
| yes | 0.57 | 0.55 | | -0.02 | | *12* | | ref. |  |
| Few rules: no | 0.47 | 0.51 | | 0.04 | | *0* | | ref. |  |
| rather no | 0.26 | 0.26 | | 0.00 | | *75* | | -0.04 | 44 |
| rather yes | 0.19 | 0.16 | | -0.03 | | *1* | | -0.10 | 5 |
| yes | 0.08 | 0.06 | | -0.02 | | *3* | | -0.08 | 26 |
| Strict rules: no | 0.14 | 0.11 | | -0.03 | | *0* | | -0.11 | 7 |
| rather no | 0.32 | 0.28 | | -0.03 | | *1* | | -0.10 | 3 |
| rather yes | 0.46 | 0.52 | | 0.06 | | *0* | | ref. |  |
| yes | 0.08 | 0.08 | | 0.00 | | *85* | | 0.06 | 38 |
| Listen to each other: no | 0.01 | 0.00 | | 0.00 | | *26* | | 0.09 | 41 |
| rather no | 0.04 | 0.04 | | 0.00 | | *91* | |  |  |
| rather yes | 0.50 | 0.52 | | 0.02 | | *19* | | ref. |  |
| yes | 0.45 | 0.44 | | -0.01 | | *33* | | 0.07 | 8 |
| missing | 0.01 | 0.00 | | 0.00 | | *10* | | ref. |  |
| Toothbrush 2 times daily | 0.77 | 0.84 | | 0.07 | | *0* | | 0.19 | 0 |
| **Regional characteristics** |  |  | |  | |  | |  |  |
| Municipality size: <5K | 0.44 | 0.36 | | -0.07 | | *0* | | 0.08 | 26 |
| 5-20K | 0.11 | 0.12 | | 0.01 | | *36* | | -0.06 | 44 |
| 20-100K | 0.27 | 0.33 | | 0.06 | | *0* | | ref. |  |
| >100K | 0.18 | 0.18 | | 0.00 | | *90* | | -0.29 | 0 |
| East * <5K |  |  | |  | |  | | -0.19 | *8* |
| East * 5-20K |  |  | |  | |  | | 0.10 | *52* |
| East * 20-100K |  |  | |  | |  | | ref. |  |
| East * >100K |  |  | |  | |  | | 0.15 | *34* |
| Recreation area | 45.62 | 37.77 | | -7.84 | | *0* | |  |  |
| East * 1. tercile | 0.16 | 0.09 | | -0.07 | | *0* | | -0.10 | *26* |
| 2. tercile | 0.16 | 0.09 | | -0.07 | | *0* | | ref. |  |
| 3. tercile | 0.17 | 0.07 | | -0.10 | | *0* | | 0.02 | *86* |
| West * 1. tercile | 0.17 | 0.26 | | 0.09 | | *0* | | -0.02 | *72* |
| 2. tercile | 0.17 | 0.25 | | 0.08 | | *0* | | ref. |  |
| 3. tercile | 0.16 | 0.25 | | 0.08 | | *0* | | -0.12 | *7* |
| Tax income/Capita | 481.2 | 569.9 | | 88.7 | | *0* | | 0.00 | *14* |
| Employed in I. Sector | 3.80 | 2.80 | | -1.00 | | *0* | | ref. |  |
| II. Sector | 34.43 | 35.54 | | 1.11 | | *1* | |  |  |
| III. Sector | 61.77 | 61.66 | | -0.11 | | *0* | | 0.00 | *1* |
| Population growth 2002-07 | -1.75 | -0.46 | | 1.29 | | *0* | | 0.01 | *23* |
| East * Population growth | -1.73 | -0.58 | | 1.15 | | *0* | | 0.00 | *94* |

Note: S1 Table 1 to be continued.

S1 Table continued

|  | No Sports | | Sports | | Sports - No Sports | | Probit Coefficient | | |
| --- | --- | --- | --- | --- | --- | --- | --- | --- | --- |
|  | Mean | Mean | | Diff. | | p-val. % | | Coef. | p-val. % |
| **Regional characteristics (continued)** |  |  | |  | |  | |  |  |
| State 1 | 0.03 | 0.03 | | 0.00 | | *29* | | -0.21 | *21* |
| State 2 | 0.01 | 0.01 | | 0.01 | | *5* | | ref. |  |
| State 3 | 0.06 | 0.11 | | 0.06 | | *0* | | 0.10 | *49* |
| State 4 | 0.14 | 0.19 | | 0.05 | | *0* | | -0.11 | *41* |
| State 5 | 0.03 | 0.06 | | 0.02 | | *0* | | -0.20 | *20* |
| State 6 | 0.04 | 0.05 | | 0.01 | | *6* | | -0.04 | *80* |
| State 7 | 0.08 | 0.14 | | 0.06 | | *0* | | -0.08 | *56* |
| State 8 | 0.12 | 0.15 | | 0.03 | | *0* | | -0.22 | *12* |
| State 9 | 0.01 | 0.01 | | 0.00 | | *47* | | ref. |  |
| State 10 | 0.03 | 0.02 | | -0.01 | | *1* | | -0.66 | *0* |
| State 11 | 0.10 | 0.06 | | -0.04 | | *0* | | -0.69 | *0* |
| State 12 | 0.07 | 0.02 | | -0.05 | | *0* | | -1.11 | *0* |
| State 13 | 0.13 | 0.06 | | -0.07 | | *0* | | -0.63 | *0* |
| State 14 | 0.07 | 0.04 | | -0.03 | | *0* | | -0.49 | *2* |
| State 15 | 0.09 | 0.04 | | -0.04 | | *0* | | -0.47 | *2* |

Note: Almost empty groups have been omitted in the estimation or have been combined with another group. Efron's R^2^ for the probit estimation is 0.21.
