# Supplementary material for: Sports and Child Development
Source: PLoS One. 2016 May 4;11(5):e0151729. doi: 10.1371/journal.pone.0151729 (PMC4856309; doi:10.1371/journal.pone.0151729)
Supplement: S2 Table — (DOCX) [file pone.0151729.s008.docx]

# S2 Table: Further estimation results – Propensity score estimation (probit) using the GCP dataset

|  | GCP A | | | | GCP B | | | | | GCP C | | | |
| --- | --- | --- | --- | --- | --- | --- | --- | --- | --- | --- | --- | --- | --- |
|  | Coef. | | | p-val. % | Coef. | | | p-val. % | | Coef. | | p-val. % | |
| Constant | 0.00 | | | *100* | -1.51 | | | *6* | | -0.62 | | *60* | |
| **Child characteristics** |  | | |  |  | | |  | |  | |  | |
| Male | 0.19 | | | *1* | 0.19 | | | *1* | | 0.25 | | *3* | |
| Age: 6 years | -0.03 | | | *74* | -0.07 | | | *53* | | -0.07 | | *64* | |
| 9 years | 0.30 | | | *0* | 0.38 | | | *15* | | 0.12 | | *73* | |
| 10 years | 0.30 | | | *0* | 0.31 | | | *24* | | -0.28 | | *39* | |
| Birthweight in grams | 0.07 | | | *54* | 0.09 | | | *46* | | 0.19 | | *32* | |
| **Child outcomes in wave 1** |  | | |  |  | | |  | |  | |  | |
| Health: Very good |  | | |  | 0.05 | | | *58* | | -0.01 | | *96* | |
| Sufficient |  | | |  | -0.18 | | | *16* | | -0.05 | | *76* | |
| Well-being: Soul |  | | |  | 0.00 | | | *25* | | 0.00 | | *49* | |
| Self |  | | |  | 0.00 | | | *98* | | 0.00 | | *68* | |
| Family |  | | |  | -0.01 | | | *8* | | 0.00 | | *51* | |
| Friends |  | | |  | 0.02 | | | *1* | | 0.00 | | *57* | |
| School |  | | |  | 0.00 | | | *23* | | 0.00 | | *38* | |
| Non-cognitive skills |  | | |  |  | | |  | |  | |  | |
| Emotional Problems |  | | |  | -0.02 | | | *62* | | 0.00 | | *100* | |
| Behavioral Problems |  | | |  | 0.02 | | | *16* | | 0.02 | | *48* | |
| Hyperactivity |  | | |  | 0.00 | | | *86* | | -0.02 | | *56* | |
| Peer Problems |  | | |  | 0.05 | | | *9* | | 0.00 | | *95* | |
| Antisocial Behavior |  | | |  | 0.01 | | | *76* | | 0.03 | | *33* | |
| **Mother's characteristics** |  | | |  |  | | |  | |  | |  | |
| Education: Basic | -0.37 | | | *0* | -0.34 | | | *0* | | -0.03 | | *82* | |
| High school | 0.06 | | | *57* | 0.04 | | | *73* | | 0.25 | | *16* | |
| University | -0.08 | | | *48* | -0.07 | | | *53* | | -0.10 | | *59* | |
| LFP: Not working | -0.03 | | | *78* | 0.06 | | | *62* | | 0.05 | | *81* | |
| Unemployed | -0.18 | | | *24* | -0.29 | | | *16* | | 0.13 | | *66* | |
| LFP: Fulltime | -0.36 | | | *0* | -0.47 | | | *0* | | -0.49 | | *1* | |
| Job: Self employed | 0.49 | | | *0* | 0.52 | | | *1* | | 0.78 | | *1* | |
| Civil servant | 0.64 | | | *1* | 0.61 | | | *3* | | 1.10 | | *2* | |
| Employed | 0.26 | | | *1* | 0.21 | | | *10* | | 0.34 | | *11* | |
| Health: Very good | -0.06 | | | *55* | -0.15 | | | *13* | | -0.22 | | *17* | |
| Ok | 0.02 | | | *87* | 0.07 | | | *48* | | 0.09 | | *56* | |
| Sufficient | 0.05 | | | *78* | -0.10 | | | *60* | | -0.19 | | *49* | |
| Smoking: Sometimes | | -0.15 | 28 | | |  |  | |  | |  | |  |
| Daily | | -0.10 | *32* | | |  |  | |  | |  | |  |

Note: S3 Table 1 to be continued.

S2 Table continued

|  | GCP A | | GCP B | | GCP C | |
| --- | --- | --- | --- | --- | --- | --- |
|  | Coef. | p-val. % | Coef. | p-val. % | Coef. | p-val. % |
| **Father's characteristics** |  |  |  |  |  |  |
| Education: Intermediate | 0.03 | *81* | 0.03 | *84* | 0.26 | *19* |
| University | -0.09 | *56* | -0.14 | *34* | 0.04 | *87* |
| Other | 0.16 | *20* | 0.11 | *39* | 0.13 | *55* |
| Missing | 0.04 | *80* | 0.71 | *1* | 0.81 | *4* |
| LFP: Not working | -0.21 | *56* |  |  |  |  |
| Unemployed | -0.49 | *0* | 0.22 | *36* | 0.06 | *86* |
| Parttime | -0.41 | *2* | -0.36 | *9* | -0.35 | *36* |
| Missing | -0.24 | *27* | 0.15 | *50* | 0.06 | *81* |
| Job: Self employed | -0.15 | *66* | -0.07 | *72* | -0.03 | *93* |
| Civil servant | -0.16 | *66* | -0.13 | *59* | -0.31 | *39* |
| Employed | -0.10 | *78* | -0.04 | *84* | -0.07 | *80* |
| Missing | -0.13 | *72* |  |  |  |  |
| Health: Very good | 0.08 | *56* | -0.05 | *68* | -0.09 | *66* |
| Ok | 0.03 | *78* | -0.13 | *27* | -0.32 | *12* |
| Sufficient | 0.17 | *43* | -0.04 | *88* | -0.20 | *65* |
| Missing | -0.17 | *9* | -0.88 | *0* | -1.01 | *0* |
| **Family characteristics** |  |  |  |  |  |  |
| Total household income | 0.00 | *5* | 0.00 | *0* | 0.00 | *4* |
| > 5.000 (binary) | 0.70 | *0* |  |  |  |  |
| Missing (binary) | 0.34 | *7* | 0.59 | *0* | 0.40 | *23* |
| Siblings in household | -0.11 | *2* | -0.18 | *0* | -0.13 | *2* |
| Older sibling in household (binary) | -0.11 | *15* | -0.07 | *40* | -0.12 | *36* |
| Single parent household | 0.08 | *74* | -0.21 | *39* | -0.04 | *89* |
| **Parenting style** |  |  |  |  |  |  |
| Smoking during pregnancy: Daily | -0.16 | *32* | -0.29 | *5* | -0.31 | *19* |
| Sometimes | -0.13 | *37* | -0.20 | *17* | -0.24 | *25* |
| Mother cares (binary) |  |  | 0.15 | *6* | 0.04 | *73* |
| Strict rule: Not at all | -0.12 | *33* |  |  |  |  |
| Rather not | 0.02 | *81* |  |  |  |  |
| Yes | 0.13 | *40* |  |  |  |  |
| Family cares: Sometimes | 0.16 | *35* |  |  |  |  |
| Always | 0.15 | *6* |  |  |  |  |

Note: S3 Table 1 to be continued.

S2 Table continued

|  | GCP A | | GCP B | | GCP C | |
| --- | --- | --- | --- | --- | --- | --- |
|  | Coef. | p-val. % | Coef. | p-val. % | Coef. | p-val. % |
| **Regional characteristics** |  |  |  |  |  |  |
| Municipality size: < 5k | 0.08 | *52* | 0.04 | *71* | -0.13 | *48* |
| 5-20k | -0.02 | *91* | -0.06 | *66* | -0.01 | *96* |
| >100k | -0.37 | *0* | -0.37 | *0* | -0.45 | *1* |
| East * Municipality size: < 5k | -0.38 | *27* | -0.20 | *58* |  |  |
| 5-20k | 0.21 | *47* | 0.30 | *32* |  |  |
| <20k |  |  |  |  | -0.09 | *82* |
| >100k | 1.08 | *0* | 1.14 | *0* | 0.55 | *21* |
| Recreation area |  |  |  |  |  |  |
| East * 1. tercile | 0.63 | *4* | 0.65 | *5* | -0.23 | *56* |
| 3. tercile | -0.01 | *98* | 0.05 | *84* | 0.09 | *79* |
| West * 1. tercile | 0.07 | *48* | -0.01 | *95* | -0.04 | *77* |
| 3. tercile | 0.26 | *1* | 0.20 | *6* | -0.02 | *90* |
| Tax income/Capita | 0.00 | *71* | 0.00 | *83* | 0.00 | *13* |
| Employed in III. Sector | 0.00 | *40* | 0.00 | *49* | 0.00 | *99* |
| Population growth 2002-07 | -0.02 | *14* | -0.03 | *13* | 0.03 | *13* |
| East | -0.96 | *0* | -1.07 | *0* | -0.60 | *6* |
| Efron's R^2^: | 0.17 |  | 0.19 |  | 0.16 |  |

Note: The columns GCP A, B, and C correspond to the respective columns in Table 7. In GCP A we use only the second wave of the GCP for both outcome and control variables, in GCP B, we con­trol additionally for the set of lagged cognitive and non-cognitive skills and replace all control va­riables by the respective control variables from wave 1. Last, we restrict the sample to children who do not participate in sports in a club in wave 1 (GCP C). Due to changes in the available va­riables between wave 1 and 2 and several almost empty groups when restricting the sample to child­ren who did not participate in sports in a club in wave 1, specifications differ slightly from each other.
