# Supplementary material for: Sports and Child Development
Source: PLoS One. 2016 May 4;11(5):e0151729. doi: 10.1371/journal.pone.0151729 (PMC4856309; doi:10.1371/journal.pone.0151729)
Supplement: S3 Table — (DOCX) [file pone.0151729.s009.docx]

# S3 Table: Further estimation results – Propensity score estimation (probit) for the semi-parametric LATE

|  | Coef. | p-val. % |
| --- | --- | --- |
| Constant | -0.57 | *63* |
| **Child characteristics** |  |  |
| Male | 0.03 | *59* |
| Age: 3 years | -0.06 | *58* |
| 4 years | 0.04 | *76* |
| 5 years | 0.05 | *64* |
| 7 years | -0.08 | *45* |
| 8 years | -0.05 | *67* |
| 9 years | -0.06 | *55* |
| 10 years | -0.01 | *91* |
| **Mother's characteristics** |  |  |
| Education: Basic | 0.07 | *45* |
| High school | -0.11 | *25* |
| University | 0.02 | *86* |
| BMI: Overweight | 0.03 | *65* |
| Obese | 0.03 | *74* |
| **Father's characteristics** |  |  |
| Education: Basic | -0.18 | *3* |
| High school | 0.10 | *38* |
| University | 0.12 | *32* |
| **Family characteristics** |  |  |
| Social class: Low | -0.10 | *19* |
| High | -0.05 | *65* |
| Single parent household | 0.22 | *4* |
| **Regional characteristics** |  |  |
| East * log Population density | 0.87 | *0* |
| log Recreation area/Capita | 0.39 | *0* |
| log Tax income/Capita | -1.00 | *0* |
| log Employed in III. Sector | 0.27 | *16* |
| West * log Population density | 0.53 | *0* |
| log Recreation area/Capita | -0.39 | *0* |
| log Tax income/Capita | -0.21 | *31* |
| log Employed in III. Sector | 0.46 | *0* |
| log Population growth 2002-07 | 16.12 | *0* |
| East | -0.24 | *86* |
| Efron's R^2^ | 0.30 |  |

Note: Estimation is based on the 'countryside' sample using the KiGGS data.
