# Supplementary material for: Sports and Child Development
Source: PLoS One. 2016 May 4;11(5):e0151729. doi: 10.1371/journal.pone.0151729 (PMC4856309; doi:10.1371/journal.pone.0151729)
Supplement: S4 Table — (DOCX) [file pone.0151729.s010.docx]

# S4 Table: Additional estimates – Comparison of matching estimates for well-being using KiGGS and GCP

|  | KiGGS | | GCP A | | GCP B | | GCP C | |
| --- | --- | --- | --- | --- | --- | --- | --- | --- |
|  | Effect | p-val. % | Effect | p-val. % | Effect | p-val. % | Effect | p-val. % |
| **Total Well-being** | **-0.05** | ***18*** | **-0.13** | ***2*** | **-0.05** | ***28*** | **-0.12** | ***16*** |
| Well-being: body | -0.06 | *11* | -0.02 | *68* | -0.02 | *81* | -0.05 | *56* |
| Well-being: soul | -0.03 | *42* | -0.16 | *2* | -0.08 | *19* | -0.12 | *17* |
| Well-being: self | -0.03 | *29* | -0.01 | *81* | 0.04 | *48* | 0.06 | *55* |
| Well-being: family | 0.09 | *1* | -0.03 | *55* | -0.03 | *65* | -0.07 | *42* |
| Well-being: friends | -0.10 | *0* | -0.12 | *0* | -0.06 | *13* | -0.13 | *5* |
| Well-being: school | -0.06 | *7* | -0.19 | *0* | -0.14 | *2* | -0.19 | *4* |

Note: The results in the first column (KiGGS) correspond to our main set of results based on the KiGGS data and displayed in Table 6. GPC A to C are based on the GCP data and display first a pure replica­tion of the KiGGS results where we use only the second wave of the GCP for both out­come and control variables (A), then the results when we control additionally for the set of lagged cogni­tive and non-cognitive skills and replace all control variables by the respective control va­riables from wave 1 (B) and last, when we repeat the strategy employed under (B) but restrict the sam­ple to children who do not participate in a sports club in wave 1. The presented effect is the aver­age treatment effect (ATE). p-values are computed by bootstrapping p-values of the t-statistic with 4999 replications.
