# Supplementary material for: Sports and Child Development
Source: PLoS One. 2016 May 4;11(5):e0151729. doi: 10.1371/journal.pone.0151729 (PMC4856309; doi:10.1371/journal.pone.0151729)
Supplement: S5 Table — (DOCX) [file pone.0151729.s011.docx]

# S5 Table: Additional estimates – Descriptive statistics for children’s cognitive and non-cognitive skills (non-standardized variables)

|  | No Sports | Sports | Sports - NoSports | | Obs. |
| --- | --- | --- | --- | --- | --- |
|  |  |  | Difference | p-val. |  |
| **Cognitive Skills** |  |  |  |  |  |
| **Overall Grade** | **2.40** | **2.19** | **-0.21** | **0** | **1698** |
| **Non-cognitive Skills** |  |  |  |  |  |
| Emotional Problems | 1.82 | 1.65 | -0.18 | 0 | 5632 |
| Behavioral Problems | 2.04 | 1.83 | -0.21 | 0 | 5632 |
| Hyperactivity | 3.51 | 3.08 | -0.43 | 0 | 5632 |
| Peer Problems | 1.42 | 1.03 | -0.38 | 0 | 5632 |
| **Overall Score** | **8.79** | **7.59** | **-1.20** | **0** | **5632** |
| Prosocial Behavior | 7.76 | 7.97 | 0.21 | 0 | 5632 |
| Note: All outcome variables are according to the original scale. For all variables except prosocial beha­vior a lower value corresponds to a better outcome. P-values stem from two-sided t-tests compar­ing the means for children doing and not doing sports in a club. | | | | | |
