# Supplementary material for: Sports and Child Development
Source: PLoS One. 2016 May 4;11(5):e0151729. doi: 10.1371/journal.pone.0151729 (PMC4856309; doi:10.1371/journal.pone.0151729)
Supplement: S6 Table — (DOCX) [file pone.0151729.s012.docx]

# S6 Table: Additional estimates – Matching estimates for cognitive and non-cognitive skills (KiGGS) (non-standardized variables)

|  | Average Outcome if Participating | Average Outcome if Not Participating | Average Effect | p-val. % |  |
| --- | --- | --- | --- | --- | --- |
| **Cognitive Skills** |  |  |  |  |  |
| **Overall Grade** | **2.21** | **2.31** | **-0.10** | ***3*** |  |
| **Non-cognitive Skills** |  |  |  |  |  |
| Emotional Problems | 1.72 | 1.89 | -0.17 | *1* |  |
| Behavioral Problems | 1.93 | 2.00 | -0.06 | *26* |  |
| Hyperactivity | 3.27 | 3.36 | -0.09 | *32* |  |
| Peer Problems | 1.09 | 1.42 | -0.33 | *0* |  |
| **Overall Score** | **8.01** | **8.66** | **-0.65** | ***0*** |  |
| Prosocial Behavior | 7.89 | 7.83 | 0.06 | *29* |  |
| Note: All outcome variables are according to the original scale. For all variables except prosocial behavior a lower value corresponds to a better outcome. p-values are computed by bootstrapping p-values of the t-statistic with 4999 replications. | | | | | |
