# Supplementary material for: Sports and Child Development
Source: PLoS One. 2016 May 4;11(5):e0151729. doi: 10.1371/journal.pone.0151729 (PMC4856309; doi:10.1371/journal.pone.0151729)
Supplement: S7 Table — (DOCX) [file pone.0151729.s013.docx]

# S7 Table: Matching estimates for cognitive and non-cognitive skills using different propensity score specifications (KiGGS)

|  | Average Outcome if Participating | Average Outcome if Not Participating | Average Effect | p-val. % |
| --- | --- | --- | --- | --- |
| **Cognitive Skills** |  |  |  |  |
| **Overall Grade** | **-0.05** | **0.13** | **-0.18** | ***1*** |
| **Non-cognitive Skills** |  |  |  |  |
| Emotional Problems | -0.03 | 0.07 | -0.10 | *0* |
| Behavioral Problems | 0.00 | 0.03 | -0.02 | *49* |
| Hyperactivity | 0.00 | 0.02 | -0.02 | *44* |
| Peer Problems | -0.09 | 0.11 | -0.19 | *0* |
| **Overall Score** | **-0.04** | **0.07** | **-0.11** | ***0*** |
| Antisocial Behavior | 0.00 | 0.02 | -0.03 | 38 |

Note: Results presented in this table resemble our main results but from an estima­tion with an alternative specification of the propensity score. In particu­lar we include interaction terms between the child's sex and age and dropped the variable height. p-values are computed by bootstrapping p-val­ues of the t-statistic with 4999 replications.
