# Supplementary material for: Sports and Child Development
Source: PLoS One. 2016 May 4;11(5):e0151729. doi: 10.1371/journal.pone.0151729 (PMC4856309; doi:10.1371/journal.pone.0151729)
Supplement: S8 Table — (DOCX) [file pone.0151729.s014.docx]

# S8 Table: Additional estimates – Matching estimates for non-cognitive skills (KiGGS - cognitive sam­ple)

|  | Y_1_ | Y_0_ | θ (ATE) | p-val. % |
| --- | --- | --- | --- | --- |
| **Non-cognitive Skills** |  |  |  |  |
| Emotional Problems | 0.03 | 0.15 | -0.12 | *5* |
| Behavioral Problems | -0.15 | -0.10 | -0.05 | *47* |
| Hyperactivity | -0.08 | -0.06 | -0.02 | *70* |
| Peer Problems | -0.05 | 0.18 | -0.23 | *0* |
| **Overall Score** | **-0.09** | **0.05** | **-0.14** | ***3*** |
| Antisocial Behavior | -0.21 | -0.06 | -0.16 | *2* |

Note: The results presented for non-cognitive skills stem from estimations based on the same sample for which school grades are available. p-values are com­puted by bootstrapping p-values of the t-statistic with 4999 replications.
