# Supplementary material for: Sports and Child Development
Source: PLoS One. 2016 May 4;11(5):e0151729. doi: 10.1371/journal.pone.0151729 (PMC4856309; doi:10.1371/journal.pone.0151729)
Supplement: S9 Table — (DOCX) [file pone.0151729.s015.docx]

# S5 Appendix: Additional estimates – Bootstrapped difference in average treatment effects between specifications with and without state fixed effects

|  | Mean Difference | Standard Error of Difference | 95% CI | |
| --- | --- | --- | --- | --- |
| **Cognitive Skills** |  |  |  |  |
| **Overall Grade** | **-0.01** | **0.05** | **-0.11** | **0.10** |
| **Non-cognitive Skills** |  |  |  |  |
| Emotional Problems | 0.00 | 0.03 | -0.06 | 0.05 |
| Behavioral Problems | 0.00 | 0.03 | -0.06 | 0.06 |
| Hyperactivity | 0.00 | 0.03 | -0.06 | 0.05 |
| Peer Problems | -0.01 | 0.03 | -0.06 | 0.05 |
| Overall Score | 0.00 | 0.03 | -0.06 | 0.05 |
| Prosocial Behavior | 0.00 | 0.03 | -0.06 | 0.05 |

Note: All statistics are computed by bootstrapping the difference of the average treatment effects of a specification with and without state fixed effects with 4999 replications.
