# Supplementary material for: Sports and Child Development
Source: PLoS One. 2016 May 4;11(5):e0151729. doi: 10.1371/journal.pone.0151729 (PMC4856309; doi:10.1371/journal.pone.0151729)
Supplement: S10 Table — (DOCX) [file pone.0151729.s016.docx]

# S9 Table: Balancing tests – After-match balancing tests (KiGGS)

|  | ATENT | | ATET | |
| --- | --- | --- | --- | --- |
|  | Std. Bias | p-val. % | Std. Bias | p-val. % |
| **Child characteristics** |  |  |  |  |
| Male | 1.55 | *58* | -0.38 | *88* |
| Age: 3 years | 0.00 | *100* | 0.06 | *98* |
| 4 years | -1.01 | *72* | -0.06 | *98* |
| 5 years | -0.31 | *91* | 0.06 | *98* |
| 7 years | 1.44 | *61* | 0.42 | *87* |
| 8 years | -1.30 | *64* | -0.57 | *82* |
| 9 years | 0.71 | *80* | 0.00 | *100* |
| 10 years | 0.59 | *83* | 0.25 | *92* |
| Height in cm | 0.20 | *94* | -0.15 | *95* |
| Birthweight in grams | 0.54 | *85* | -0.50 | *84* |
| **Mother's characteristics** |  |  |  |  |
| Education: Basic | 1.57 | *58* | -2.09 | *41* |
| High school | 1.40 | *62* | 0.53 | *84* |
| University | -1.81 | *52* | -0.33 | *90* |
| Other | 3.10 | *27* | -0.20 | *94* |
| LFP: Not working | 4.11 | *14* | 0.69 | *79* |
| Unemployed | -1.40 | *62* | -0.58 | *82* |
| Maternal leave | 1.52 | *59* | 0.10 | *97* |
| Fulltime | -2.35 | *40* | -1.55 | *54* |
| Job: Unskilled | 0.36 | *90* | -3.26 | *20* |
| Highskilled | -0.87 | *76* | -0.63 | *80* |
| Self employed | -0.35 | *90* | -0.16 | *95* |
| Housewife | 1.91 | *50* | 0.98 | *70* |
| Underweight | -0.66 | *81* | 1.66 | *52* |
| Overweight | -0.43 | *88* | -3.05 | *23* |
| Obese | 0.78 | *78* | 0.13 | *96* |
| **Father's characteristics** |  |  |  |  |
| Education: Basic | -2.17 | *44* | -3.22 | *21* |
| High school | 1.68 | *55* | 1.53 | *55* |
| University | -1.34 | *63* | 1.34 | *60* |
| Other | 1.57 | *58* | -1.88 | *46* |
| LFP: Not working | -0.70 | *80* | -0.87 | *73* |
| Unemployed | 3.80 | *18* | 0.26 | *92* |
| Parttime | -8.40 | *0* | -0.10 | *97* |
| Job: Unskilled job | -0.99 | *73* | -0.71 | *78* |
| Highskilled job | 0.52 | *85* | 0.53 | *84* |
| Self employed | -0.69 | *81* | -0.40 | *88* |
| Overweight | -1.11 | *69* | -0.86 | *74* |
| Obese | -0.77 | *78* | 0.47 | *85* |
| Missing | 1.23 | *66* | 0.48 | *85* |

Note: S9 Table to be continued.

S9 Table continued

|  | *ATENT* | | *ATET* | |
| --- | --- | --- | --- | --- |
|  | Std. Bias | p-val. % | Std. Bias | p-val. % |
| **Family characteristics** |  |  |  |  |
| Social class: Low | 0.00 | *100* | 0.00 | *100* |
| High | -0.07 | *98* | 0.88 | *73* |
| Total household income | 0.76 | *79* | -0.34 | *89* |
| > 5000 (binary) | -0.04 | *99* | 2.19 | *39* |
| Missing (binary) | 0.49 | *86* | -1.88 | *46* |
| Single parent household | 1.01 | *72* | -0.83 | *74* |
| Siblings in household | 6.85 | *2* | -0.84 | *74* |
| Older sibling in hh (binary) | 2.94 | *30* | -2.00 | *43* |
| Mold at home | -1.13 | *69* | 1.94 | *45* |
| **Parenting style** |  |  |  |  |
| Smoking during pregnancy: regularly | 6.67 | *2* | 0.14 | *96* |
| occasionally | -1.29 | *65* | 0.74 | *77* |
| Family cares: no/rather no | -1.56 | *58* | -0.66 | *80* |
| rather yes | 0.45 | *87* | -6.04 | *2* |
| Few rules: rather no | -2.39 | *40* | -0.62 | *81* |
| rather yes | -2.03 | *47* | 0.42 | *87* |
| yes | 5.00 | *8* | -1.52 | *55* |
| Strict rules: no | 0.33 | *91* | 1.85 | *47* |
| rather no | 0.43 | *88* | -3.23 | *21* |
| yes | -2.00 | *48* | -0.06 | *98* |
| Listen to each other: no/rahter no | -3.11 | *27* | -0.12 | *96* |
| yes | -0.34 | *90* | 3.68 | *15* |
| Toothbrush 2 times daily | 0.12 | *96* | 1.04 | *68* |
| **Regional characteristics** |  |  |  |  |
| Municipality size: <5K | -1.84 | *51* | -2.66 | *30* |
| 5-20K | 3.27 | *25* | -1.35 | *60* |
| >100K | -0.51 | *86* | 1.51 | *55* |
| East * <5K | -2.79 | *32* | -0.73 | *78* |
| East * 5-20K | 1.70 | *55* | -0.77 | *76* |
| East * >100K | -2.58 | *36* | 1.02 | *69* |
| East * 1. tercile | -4.95 | *8* | -0.32 | *90* |
| 3. tercile | 3.23 | *25* | 0.29 | *91* |
| West * 1. tercile | 1.86 | *51* | 0.63 | *81* |
| 3. tercile | 0.10 | *97* | 0.85 | *74* |
| Tax income/Capita | 0.23 | *93* | 0.19 | *94* |
| III. Sector | -0.19 | *95* | 0.53 | *84* |
| Population growth 2002-07 | -1.68 | *55* | -0.75 | *77* |
| East * Population growth | -1.08 | *70* | -1.48 | *56* |

Note: S9 Table to be continued.

S9 Table continued

|  | *ATENT* | | | | | *ATET* | | |
| --- | --- | --- | --- | --- | --- | --- | --- | --- |
|  | Std. Bias | | | p-val. % | | Std. Bias | | p-val. % |
| State 1 | | 0.51 | *86* | | -0.74 | | *77* | |
| State 3 | | 0.79 | *78* | | 0.55 | | *83* | |
| State 4 | | 0.92 | *74* | | 0.58 | | *82* | |
| State 5 | | 0.24 | *93* | | 4.35 | | *9* | |
| State 6 | | 1.61 | *57* | | -1.69 | | *51* | |
| State 7 | | 2.16 | *44* | | -3.68 | | *15* | |
| State 8 | | -0.34 | *90* | | 1.03 | | *69* | |
| State 10 | | -0.32 | *91* | | 0.73 | | *77* | |
| State 11 | | -1.83 | *51* | | -1.51 | | *55* | |
| State 12 | | 5.22 | *6* | | -0.84 | | *74* | |
| State 13 | | -1.89 | *50* | | 1.04 | | *69* | |
| State 14 | | -3.17 | *26* | | 0.35 | | *89* | |
| State 15 | | -4.19 | *14* | | 0.83 | | *75* | |
|  |  | | |  | |  | |  |
| Joint test for imbalance (χ^2^-statistic) | 0.10 | | | *76* | | 0.00 | | *95* |

Note: p-values of 2-sample t-tests. Std. Bias: Standardized bias.
