# Supplementary material for: Sports and Child Development
Source: PLoS One. 2016 May 4;11(5):e0151729. doi: 10.1371/journal.pone.0151729 (PMC4856309; doi:10.1371/journal.pone.0151729)
Supplement: S11 Table — (DOCX) [file pone.0151729.s017.docx]

# S10 Table: Balancing tests – After-match balancing tests (GCP)

|  | GCP A | | | | GCP B | | | | GCP C | | | |
| --- | --- | --- | --- | --- | --- | --- | --- | --- | --- | --- | --- | --- |
|  | ATENT | | ATET | | ATENT | | ATET | | ATENT | | ATET | |
|  | Std. Bias | p-val. % | Std. Bias | p-val. % | Std. Bias | p-val. % | Std. Bias | p-val. % | Std. Bias | p-val. % | Std. Bias | p-val. % |
| **Child characteristics** |  |  |  |  |  |  |  |  |  |  |  |  |
| Male | -1.52 | *81* | -3.59 | *45* | -3.44 | *59* | -3.44 | *59* | -2.65 | *79* | -2.65 | *79* |
| Age: 6 years | 4.82 | *45* | 2.53 | *59* | 0.71 | *91* | 0.71 | *91* | 1.88 | *85* | 1.88 | *85* |
| Age: 9 years | 1.83 | *77* | 0.01 | *100* | 5.59 | *38* | 5.59 | *38* | -2.17 | *82* | -2.17 | *82* |
| Age: 10 years | 1.23 | *85* | 0.49 | *92* | -3.83 | *55* | -3.83 | *55* | 1.03 | *92* | 1.03 | *92* |
| Birthweight in grams | -2.13 | *74* | 4.70 | *32* | 1.91 | *76* | 1.91 | *76* | 0.84 | *93* | 0.84 | *93* |
| **Child outcomes in wave 1** |  |  |  |  |  |  |  |  |  |  |  |  |
| Health: Very good |  |  |  |  | 1.31 | *84* | 1.31 | *84* | -8.46 | *39* | -8.46 | *39* |
| Health: Sufficient |  |  |  |  | 6.62 | *30* | 6.62 | *30* | -4.55 | *64* | -4.55 | *64* |
| Well-being: Soul |  |  |  |  | -4.53 | *48* | -4.53 | *48* | -1.74 | *86* | -1.74 | *86* |
| Well-being: Self |  |  |  |  | -0.01 | *100* | -0.01 | *100* | 7.44 | *45* | 7.44 | *45* |
| Well-being: Family |  |  |  |  | -0.16 | *98* | -0.16 | *98* | 4.90 | *62* | 4.90 | *62* |
| Well-being: Friends |  |  |  |  | 1.09 | *86* | 1.09 | *86* | 0.67 | *94* | 0.67 | *94* |
| Well-being: School |  |  |  |  | 0.81 | *90* | 0.81 | *90* | 2.36 | *81* | 2.36 | *81* |
| Emotional Problems |  |  |  |  | 4.68 | *46* | 4.68 | *46* | -0.55 | *96* | -0.55 | *96* |
| Behavioral Problems |  |  |  |  | -0.88 | *89* | -0.88 | *89* | -6.74 | *49* | -6.74 | *49* |
| Hyperactivity |  |  |  |  | 4.45 | *48* | 4.45 | *48* | 1.28 | *90* | 1.28 | *90* |
| Peer Problems |  |  |  |  | -3.81 | *55* | -3.81 | *55* | -3.97 | *68* | -3.97 | *68* |
| Antisocial Behavior |  |  |  |  | -0.76 | *91* | -0.76 | *91* | 2.89 | *77* | 2.89 | *77* |
| **Mother's characteristics** |  |  |  |  |  |  |  |  |  |  |  |  |
| Education: Basic | -8.27 | *19* | -1.15 | *81* | 2.97 | *64* | 2.97 | *64* | 8.28 | *40* | 8.28 | *40* |
| Education: High school | 0.42 | *95* | 2.83 | *55* | -2.75 | *67* | -2.75 | *67* | -1.50 | *88* | -1.50 | *88* |
| Education: University | 2.09 | *74* | -1.41 | *77* | -7.82 | *22* | -7.82 | *22* | -1.02 | *92* | -1.02 | *92* |
| LFP: Not working | 3.32 | *60* | -6.35 | *18* | 1.04 | *87* | 1.04 | *87* | -1.02 | *92* | -1.02 | *92* |
| LFP: Unemployed | -6.69 | *29* | -0.68 | *89* | 1.83 | *77* | 1.83 | *77* | -3.87 | *69* | -3.87 | *69* |
| LFP: Fulltime | 0.81 | *90* | 2.35 | *62* | 1.33 | *83* | 1.33 | *83* | 5.92 | *54* | 5.92 | *54* |
| Job: Self employed | 3.38 | *59* | 1.20 | *80* | -2.49 | *70* | -2.49 | *70* | -4.92 | *61* | -4.92 | *61* |
| Job: Civil servant | -0.76 | *91* | 0.30 | *95* | -6.77 | *29* | -6.77 | *29* | -1.23 | *90* | -1.23 | *90* |
| Job: Employed | -5.44 | *39* | 4.23 | *37* | 3.32 | *60* | 3.32 | *60* | 8.87 | *36* | 8.87 | *36* |
| Health: Very good | 2.26 | *72* | 0.42 | *93* | 5.90 | *35* | 5.90 | *35* | -0.04 | *100* | -0.04 | *100* |
| Health: Ok | 3.40 | *59* | -4.33 | *36* | -1.20 | *85* | -1.20 | *85* | 6.89 | *48* | 6.89 | *48* |
| Health: Sufficient | 1.80 | *78* | 0.70 | *88* | -1.85 | *77* | -1.85 | *77* | 6.00 | *54* | 6.00 | *54* |
| Smoking: Sometimes | -0.23 | *97* | -5.60 | *24* |  |  |  |  |  |  |  |  |
| Smoking: Daily | 0.36 | *96* | -1.19 | *80* |  |  |  |  |  |  |  |  |
| **Father's characteristics** |  |  |  |  |  |  |  |  |  |  |  |  |
| Education: Intermediate | 0.68 | *92* | -0.64 | *89* | -1.52 | *81* | -1.52 | *81* | 3.10 | *75* | 3.10 | *75* |
| Education: University | -7.97 | *21* | -2.03 | *67* | -5.37 | *40* | -5.37 | *40* | 3.36 | *73* | 3.36 | *73* |
| Education: Other | 0.10 | *99* | -3.32 | *48* | -1.23 | *85* | -1.23 | *85* | -12.40 | *20* | -12.40 | *20* |
| Education: Missing | -3.23 | *61* | 2.59 | *58* | 6.26 | *33* | 6.26 | *33* | 4.35 | *66* | 4.35 | *66* |
| LFP: Not working | -2.49 | *69* | -2.47 | *60* |  |  |  |  |  |  |  |  |
| LFP: Unemployed | 0.28 | *97* | 0.22 | *96* | 2.88 | *65* | 2.88 | *65* | -2.25 | *82* | -2.25 | *82* |
| LFP: Parttime | 3.61 | *57* | -0.91 | *85* | -0.57 | *93* | -0.57 | *93* | -8.30 | *39* | -8.30 | *39* |
| LFP: Missing | -2.68 | *67* | 4.01 | *40* | -4.44 | *49* | -4.44 | *49* | -1.13 | *91* | -1.13 | *91* |

Note: S10 Table to be continued.

S10 Table continued

|  | GCP A | | | | GCP B | | | | GCP C | | | |
| --- | --- | --- | --- | --- | --- | --- | --- | --- | --- | --- | --- | --- |
|  | ATENT | | ATET | | ATENT | | ATET | | ATENT | | ATET | |
|  | Std. Bias | p-val. % | Std. Bias | p-val. % | Std. Bias | p-val. % | Std. Bias | p-val. % | Std. Bias | p-val. % | Std. Bias | p-val. % |
| Job: Self employed | 1.28 | *84* | 0.37 | *94* | -2.91 | *65* | -2.91 | *65* | 0.38 | *97* | 0.38 | *97* |
| Job: Civil servant | 1.92 | *76* | -3.94 | *41* | -1.33 | *83* | -1.33 | *83* | -0.39 | *97* | -0.39 | *97* |
| Job: Employed | 2.32 | *72* | -1.45 | *76* | -1.99 | *75* | -1.99 | *75* | -2.14 | *83* | -2.14 | *83* |
| Job: Missing | -3.64 | *57* | 3.11 | *51* |  |  |  |  |  |  |  |  |
| Health: Very good | -0.36 | *95* | -5.46 | *25* | 0.82 | *90* | 0.82 | *90* | -13.21 | *18* | -13.21 | *18* |
| Health: Ok | 0.93 | *88* | 2.91 | *54* | -2.17 | *73* | -2.17 | *73* | 3.65 | *71* | 3.65 | *71* |
| Health: Sufficient | -1.03 | *87* | 3.15 | *51* | -7.02 | *27* | -7.02 | *27* | -0.60 | *95* | -0.60 | *95* |
| Health: Missing | 0.78 | *90* | -0.18 | *97* | 7.15 | *26* | 7.15 | *26* | 4.25 | *66* | 4.25 | *66* |
| **Family characteristics** |  |  |  |  |  |  |  |  |  |  |  |  |
| Total hh income | -1.22 | *85* | 0.03 | *100* | -1.80 | *78* | -1.80 | *78* | -0.78 | *94* | -0.78 | *94* |
| Total hh income missing (binary) | -0.24 | *97* | -2.94 | *53* | 1.25 | *84* | 1.25 | *84* | 6.80 | *49* | 6.80 | *49* |
| Total hh income > 5.000 | 3.72 | *56* | 2.72 | *57* |  |  |  |  |  |  |  |  |
| Siblings in household | 9.83 | *12* | -2.12 | *66* | 2.19 | *73* | 2.19 | *73* | -5.00 | *61* | -5.00 | *61* |
| Older sibling in hh (binary) | 2.76 | *66* | -3.39 | *47* | -1.08 | *87* | -1.08 | *87* | 2.47 | *80* | 2.47 | *80* |
| Single parent household | -7.68 | *23* | 3.92 | *41* | -1.69 | *79* | -1.69 | *79* | -0.38 | *97* | -0.38 | *97* |
| **Parenting style** |  |  |  |  |  |  |  |  |  |  |  |  |
| Smoking during pregnancy: daily | 0.94 | *88* | 0.99 | *83* | 2.34 | *71* | 2.34 | *71* | 3.97 | *68* | 3.97 | *68* |
| Smoking during pregnancy: sometimes | -0.17 | *98* | -1.54 | *74* | -3.84 | *55* | -3.84 | *55* | -4.80 | *62* | -4.80 | *62* |
| Mother cares (binary) |  |  |  |  | -0.93 | *88* | -0.93 | *88* | -1.21 | *90* | -1.21 | *90* |
| Strict rule: not at all | -0.36 | *96* | 1.44 | *76* |  |  |  |  |  |  |  |  |
| rather not | -4.38 | *49* | -4.64 | *33* |  |  |  |  |  |  |  |  |
| yes | 0.85 | *89* | 1.35 | *78* |  |  |  |  |  |  |  |  |
| Family talks with child: sometimes | 2.84 | *65* | -3.20 | *50* |  |  |  |  |  |  |  |  |
| always | -4.32 | *50* | -1.13 | *81* |  |  |  |  |  |  |  |  |
| **Regional characteristics** |  |  |  |  |  |  |  |  |  |  |  |  |
| Municipality size: < 5k | -3.93 | *54* | 0.01 | *100* | -1.24 | *85* | -1.24 | *85* | 0.64 | *95* | 0.64 | *95* |
| Municipality size: 5-20k | 5.61 | *38* | 0.65 | *89* | 2.34 | *71* | 2.34 | *71* | 4.85 | *62* | 4.85 | *62* |
| Municipality size: >100k | 1.25 | *84* | 1.26 | *79* | -2.94 | *64* | -2.94 | *64* | -3.54 | *72* | -3.54 | *72* |
| East: Municipality size: < 5k | -7.03 | *27* | 0.85 | *86* | -4.90 | *44* | -4.90 | *44* |  |  |  |  |
| East: Municipality size: 5-20k | 3.96 | *53* | 0.11 | *98* | 0.94 | *88* | 0.94 | *88* |  |  |  |  |
| East: Municipality size: <20k |  |  |  |  |  |  |  |  | 0.27 | *98* | 0.27 | *98* |
| East: Municipality size: >100k | 2.39 | *71* | -0.81 | *86* | 6.47 | *31* | 6.47 | *31* | 2.81 | *77* | 2.81 | *77* |
| East: Recreation area- first tercile | -4.93 | *44* | 0.41 | *93* | -1.36 | *83* | -1.36 | *83* | -1.72 | *86* | -1.72 | *86* |
| East: Recreation area- third tercile | 3.52 | *58* | -0.94 | *84* | -2.41 | *70* | -2.41 | *70* | 2.28 | *82* | 2.28 | *82* |
| West: Recreation area- first tercile | -0.82 | *90* | -8.10 | *9* | -4.52 | *48* | -4.52 | *48* | -3.13 | *75* | -3.13 | *75* |
| West: Recreation area- third tercile | -1.07 | *87* | 4.64 | *33* | -0.59 | *93* | -0.59 | *93* | 1.73 | *86* | 1.73 | *86* |
| Tax income/Capita | -0.89 | *89* | 1.33 | *78* | -1.75 | *78* | -1.75 | *78* | -3.80 | *70* | -3.80 | *70* |
| Employed in III. Sector | 0.42 | *95* | 1.11 | *82* | -2.63 | *68* | -2.63 | *68* | -4.24 | *66* | -4.24 | *66* |
| Population growth 2002-07 | 6.28 | *32* | 1.45 | *76* | -3.48 | *58* | -3.48 | *58* | -6.46 | *51* | -6.46 | *51* |
| East | 0.00 | *100* | 0.00 | *100* | 0.00 | *100* | 0.00 | *100* | 0.25 | *98* | 0.25 | *98* |
|  |  |  |  |  |  |  |  |  |  |  |  |  |
| Joint test for imbalance (χ^2^-statistic) | 0.05 | *82* | 0.09 | *76* | 0.16 | *69* | *0* | *70* | 0.02 | *88* | 0.00 | *99* |

Note: p-values of 2-sample t-tests. Std. Bias: Standardized bias.
