# Supplementary material for: Sports and Child Development
Source: PLoS One. 2016 May 4;11(5):e0151729. doi: 10.1371/journal.pone.0151729 (PMC4856309; doi:10.1371/journal.pone.0151729)
Supplement: S12 Table — (DOCX) [file pone.0151729.s018.docx]

# S12 Table: Balancing tests – After-match balance tests for semi-parametric IV

|  | ATENT | | ATET | |
| --- | --- | --- | --- | --- |
|  | Std. Bias | p-val. % | Std. Bias | p-val. % |
| **Child characteristics** |  |  |  |  |
| Male | -6.05 | *21* | -1.53 | *64* |
| Age: 3 years | 0.79 | *87* | 1.40 | *67* |
| Age: 4 years | -0.77 | *87* | -2.51 | *44* |
| Age: 5 years | -0.25 | *96* | -0.99 | *76* |
| Age: 7 years | -1.73 | *72* | -1.43 | *66* |
| Age: 8 years | 1.24 | *80* | -1.75 | *59* |
| Age: 9 years | -1.94 | *69* | -1.48 | *65* |
| Age: 10 years | 2.25 | *64* | 2.24 | *50* |
| **Mother's characteristics** |  |  |  |  |
| Education: Basic | -2.91 | *55* | -0.94 | *77* |
| Education: High school | -4.02 | *41* | -1.24 | *71* |
| Education: University | 0.52 | *91* | 5.56 | *9* |
| BMI: Overweight | 1.81 | *71* | 4.01 | *22* |
| BMI: Obese | -1.36 | *78* | -4.12 | *21* |
| **Father's characteristics** |  |  |  |  |
| Education: Basic | -3.80 | *43* | -6.03 | *7* |
| Education: High school | -9.63 | *5* | 0.16 | *96* |
| Education: University | -0.86 | *86* | 6.66 | *4* |
| **Family characteristics** |  |  |  |  |
| Social class: Low | 0.00 | *100* | 0.00 | *100* |
| Social class: High | -1.05 | *83* | 5.97 | *7* |
| Single parent household | -2.42 | *62* | 4.27 | *19* |
| **Regional characteristics** |  |  |  |  |
| East: log population density | 4.22 | *38* | 10.74 | *0* |
| East: log recreation area per capita (in m^2)^ | -8.34 | *9* | 4.91 | *13* |
| East: log tax income per capita | 0.63 | *90* | 7.34 | *3* |
| East: log share of labor force in tertiary sector | -1.04 | *83* | 7.80 | *2* |
| West: log population density | 0.08 | *99* | -7.72 | *2* |
| West: log recreation area per capita (in m^2^) | 2.24 | *64* | -6.85 | *4* |
| West: log tax income per capita | -0.28 | *95* | -7.35 | *3* |
| West: log share of labor force in tertiary sector | 0.55 | *91* | -7.02 | *3* |
| West: log population change | -1.16 | *81* | -7.60 | *2* |
| East | 0.10 | *98* | 7.42 | *2* |
|  |  |  |  |  |
| Joint test for imbalance (χ^2^-statistic) | 46.59 | *2* | 63.88 | *0* |

Note: p-values of 2-sample t-tests. Std. Bias: Standardized bias.
